# Genome-wide association study and development of molecular markers for yield and quality traits in peanut (*Arachis hypogaea* L.)

**DOI:** 10.1186/s12870-024-04937-5

**Published:** 2024-04-05

**Authors:** Minjie Guo, Li Deng, Jianzhong Gu, Jianli Miao, Junhua Yin, Yang Li, Yuanjin Fang, Bingyan Huang, Ziqi Sun, Feiyan Qi, Wenzhao Dong, Zhenhua Lu, Shaowei Li, Junping Hu, Xinyou Zhang, Li Ren

**Affiliations:** 1 Peanut Institute, Kaifeng Academy of Agricultural and Forestry Sciences, Kaifeng, 475004 China; 2 Shennong Laboratory, Henan Provincial Key Laboratory for Oil Crops Improvement, Henan Academy of Crop Molecular Breeding, Henan Academy of Agricultural Sciences, Zhengzhou, 450002 China

**Keywords:** Peanut, Yield traits, Quality traits, Re-sequencing, Genome-wide association study (GWAS), Kompetitive allele-specific PCR (KASP) marker

## Abstract

**Background:**

This study aims to decipher the genetic basis governing yield components and quality attributes of peanuts, a critical aspect for advancing molecular breeding techniques. Integrating genotype re-sequencing and phenotypic evaluations of seven yield components and two grain quality traits across four distinct environments allowed for the execution of a genome-wide association study (GWAS).

**Results:**

The nine phenotypic traits were all continuous and followed a normal distribution. The broad heritability ranged from 88.09 to 98.08%, and the genotype-environment interaction effects were all significant. There was a highly significant negative correlation between protein content (PC) and oil content (OC). The 10× genome re-sequencing of 199 peanut accessions yielded a total of 631,988 high-quality single nucleotide polymorphisms (SNPs), with 374 significant SNP loci identified in association with the nine traits of interest. Notably, 66 of these pertinent SNPs were detected in multiple environments, and 48 of them were linked to multiple traits of interest. Five loci situated on chromosome 16 (Chr16) exhibited pleiotropic effects on yield traits, accounting for 17.64–32.61% of the observed phenotypic variation. Two loci on Chr08 were found to be strongly associated with protein and oil contents, accounting for 12.86% and 14.06% of their respective phenotypic variations, respectively. Linkage disequilibrium (LD) block analysis of these seven loci unraveled five nonsynonymous variants, leading to the identification of one yield-related candidate gene and two quality-related candidate genes. The correlation between phenotypic variation and SNP loci in these candidate genes was validated by Kompetitive allele-specific PCR (KASP) marker analysis.

**Conclusions:**

Overall, molecular markers were developed for genetic loci associated with yield and quality traits through a GWAS investigation of 199 peanut accessions across four distinct environments. These molecular tools can aid in the development of desirable peanut germplasm with an equilibrium of yield and quality through marker-assisted breeding.

**Supplementary Information:**

The online version contains supplementary material available at 10.1186/s12870-024-04937-5.

## Background

Cultivated peanut (*Arachis hypogaea* L.) is a significant legume crop that is widely grown in over one hundred countries across Asia, Africa, and North and South America. In 2021, the global peanut planting area was 32.72 million hectares, and the total peanut production amounted to 53.93 million tons [1]. Peanuts are a vital source of edible vegetable oil for human consumption. As living standards improve and consumption habits become more refined, there is an expected increase in demand for premium vegetable oils, including peanut oil. In recent years, breeding new peanut varieties with enhanced yield and improved quality has become the primary strategy for expanding the global peanut business. Unlike the pre-genomic era, peanut breeders today have access to a plethora of innovative technologies for genetic improvement. One of these methods is genome-wide association study (GWAS), which employ linkage disequilibrium (LD) to detect gene loci and their allelic variations in natural populations, linking allelic variations with target traits to analyze gene effects [2]. Since its inception in plants in 2001, GWAS has been used as a major tool for identifying genetic loci associated with traits of interest. In peanuts and other crops, GWAS has effectively identified significant loci controlling major agronomic traits, not least with respect to yield components and quality attributes that have complex genetic underpinnings involving the interaction of multiple alleles and loci that are only partially understood [3–7].

Peanut yield components, such as hundred-pod weight (HPW), hundred-seed weight (HSW), pod number per plant (PN), seed number per plant (SN), pod length (PL), and pod width (PW), are each regulated by their own genetic program while also being intricately related to one another [8]. In recent years, several yield trait QTLs have been reported for peanuts. For example, using SSR (simple sequence repeats) markers, multiple QTL related to several yield components, including HPW, PL, and PW, were identified in a BC_2_F_2:3_ population [9], and two major QTLs associated with HPW were identified in a different F_2:3_ population [10]. Based on a population composed of mutants, 58 markers associated with 39 yield and quality traits were identified using 110 *Arachis hypogaea* transferable element (AhTE) markers [11]. In several recombination inbred line (RIL) populations, QTLs for HPW and HSW were detected in multiple growth environments [12–14]. More recently, with the release of the reference genome of cultivated peanut [15], single nucleotide polymorphism (SNP)-based QTLs related to HSW, SL (seed length), and SW (seed width) were found on chromosomes A02, A05, A06, and B06 [16–20].

Protein and oil, which together account for around 75% of the peanut kernel, are the two primary storage compounds in peanuts [21]. The relative proportion of oil and protein determines the quality of a peanut and varies depending on consumer preference and intended uses. Seven QTLs related to protein content with phenotypic variation explained (PVE) ranging from 1.5 to 10.70% have been reported [22]. Additionally, 78 QTLs associated with oil content were found in two RIL populations [23], and another study identified 20 QTLs related to quality traits on chromosomes A02, A05, A07-A10, B01, B04, and B09 [24]. Yield and quality traits were analyzed in a RIL population using genotyping-by-sequencing, AhTE and SSR markers, and it was found that the QTLs for yield and protein traits were located on A02 and B06, respectively [25]. More recent research efforts have identified an oil content-related QTL (*qOCA08.1*) in a 0.8 Mb region on chromosome A08 and a crucial QTL (*qAh05.1*) that influences both oil and protein contents [26, 27]. These findings highlight that the loci impacting yield components cluster on chromosomes A02, A05, A06, A07, B05, and B06, while those governing quality attributes were located on chromosomes A05, A07, A08, A09, B01, B04, B06 and B09. Using diverse population materials to identify new loci is critical for gaining a more nuanced knowledge of the genetic basis of peanut yield and quality attributes.

With the rapid development of sequencing technology, particularly the release of the whole genome sequence of cultivated peanuts [15, 28, 29], a large number of SNPs can be obtained using whole genome scanning technology. This presents a promising opportunity to rapidly explore the genetic basis of yield and quality traits, identify key genes, and incorporate them into peanut breeding programs. Previous research on important peanut traits mainly used artificially constructed segregation populations, GBS (genotyping-by-sequencing), and SNP chips. However, the results of such research on peanut yield and quality traits varied, and most of the candidate genes were not identified or investigated. GWAS, in contrast, can identify more allele variations by leveraging natural populations, abrogating the time-consuming and labor-intensive process of population construction. Significant locus verification and GWAS analysis using re-sequencing data are still in their early stages.

This study employed GWAS by utilizing 199 accessions, comprising both released varieties and advanced breeding lines derived from the Kainong breeding program, alongside re-sequencing and phenotypic data gathered over three years in four distinct environments. The results not only shed more light on the genetic basis of yield and quality traits but also offer valuable insights for cloning and characterizing the underlying genes as well as developing molecular markers. These markers can facilitate the molecular breeding and genetic engineering of novel peanut germplasm with enhanced yield potential and desirable quality traits.

## Materials and methods

### Plant materials and growth conditions

A total of 199 Chinese peanut germplasm accessions were collected from various sites across China, including the Kaifeng Academy of Agricultural and Forestry Sciences in Henan province, the Hebei Academy of Agriculture and Forestry Sciences, and the Institute of Oil Crops at the Chinese Academy of Agricultural Sciences in Hubei province. Some accessions have been registered by the state, while others are advanced breeding lines (Fig. S1; Dataset S1). The selected 199 lines were cultivated over three consecutive years (2019, 2020, and 2021) at experimental fields in Kaifeng (114°27′E, 34°77′N) and Xinyang (114°07′E, 32°12′N) in Henan province, China. Four experimental environments were designated as E1 (Kaifeng in 2019), E2 (Xinyang in 2019), E3 (Kaifeng in 2020), and E4 (Kaifeng in 2021). A randomized block design with three replicates was employed, planting 165 seeds of each material in a 6.67 × 2 m^2^ plot [30]. Each row contained 33 plants per accession, with a 40 cm spacing between rows and a 20 cm spacing between individual plants. The experimental field featured medium soil fertility, good drainage and irrigation, level topography, and sandy loam. After harvesting, seven yield components, HPW, HSW, SP, NP (total number of 500 g of pods), NS (total number of 250 g of seeds), PL, and PW, were measured according to the standard procedures [31]. Quality traits, including protein content (PC) and oil content (OC), were determined using near-infrared reflectance spectroscopy (DA7250; Perten Instruments, Beijing, China).

### DNA extraction and genotype sequencing

Genomic DNA was extracted from approximately 100 mg of unfolded leaves collected from 3-week-old seedlings using a plant genomic DNA kit (Tiangen, Beijing, China). DNA integrity, quality, and concentration were assessed through gel electrophoresis, NanoDrop™2000 (Thermo Fisher, Waltham, MA), and a Qubit fluorometer (Thermo Fisher). Qualified DNA samples were randomly fragmented with a Covaris® ultrasonic breaker (Covaris, Woburn, MA) before library construction. The process included end-repairing and phosphorylation, A-tailing, index adapter ligation, denaturation, and PCR amplification. The constructed library was sequentially sequenced on an Illumina HiSeq^TM^2000 platform (Illumina, San Diego, CA) by Novogene (Beijing, China).

### Analysis of phenotypic data

Phenotypic statistical analyses were conducted using mixed linear models in Genstat version 22.0 [32] using Kainong 69 as a control. Phenotype summary statistics and correlation analysis were performed using DPS v20.0 and the performance analytics package of the R language [33]. The variance component was analyzed by calculating the generalized heritability of phenotypes using restricted maximum likelihood (REML) and the following formula: h^2^ = σ^2^_g_/(σ^2^_g_ + σ^2^_ge_/n + σ^2^_ε_ /nr), where σ^2^_g_ represents the genotypic variance, σ^2^_ge_ represents the interaction between 199 genotypes and the environment; σ^2^_ε_ represents the residual variance component; n is the number of environment trials; and r is the number of replicates in each environment trial [34].

### SNP alignment and calling

Paired-end re-sequencing reads were mapped to the reference genome of *A. hypogaea* Kaixuan 016 (in preparation for publication) using the Burrows-Wheeler Aligner software version 0.7.8 [35]. After sorting, potential PCR duplicates were removed by ‘rmdup’. When multiple read pairs with identical external coordinates were found, only the pair with the highest mapping quality was retained [36]. Following alignment, population-scale SNP calling was performed using SAMtools [35]. To abate SNP calling errors caused by incorrect mapping or insertions and deletions (InDels), only high-quality SNPs (coverage depth ≥ 3, RMS mapping quality ≥ 20, maf ≥ 0.05, miss ≤ 0.2) were used for subsequent analysis. The density of SNP loci was statistically analyzed using the R language.

### Linkage disequilibrium and population genetic structure

LD decay analysis was conducted using PopLDdecay software [37]. Population structure was analyzed using Admixture 1.23 software [38]. To clarify the phylogenetic relationship from a genome-wide perspective, an individual-based neighbor-joining (NJ) tree was constructed based on the *p*-distance using TreeBest 1.9.2 software (http://treesoft.sourceforge.net/treebest.shtml) and visualized using MEGA6.0 [39]. Genetic structure was evaluated by PCA using GCTA 1.24.2 software [40], and the significance level of the eigenvectors was determined using the Tracey-Widom test [41]. Heatmaps were drawn using kinship analysis in the TASSEL software package [42].

### Genome-wide association analysis and candidate gene discovery

GWAS was conducted using GEMMA (genome-wide efficient mixed-model association) version 0.94.1 [43, 44]. The mixed linear model (MLM) analysis was performed using the following equation:

*y* = *Xα* + *Sβ* + *Kµ* + *e*.

In this equation, y corresponds to phenotype; X corresponds to genotype; S represents the structure matrix, and K represents the relative kinship matrix. *Xα* and *Sβ* represent fixed effects, while *Kµ* and e represent random effects. The top three principal components were used to construct the S matrix for population structure correction. The matrix of simple matching coefficients was used to build the K matrix. Annotation was performed using the ANOVAR package against the reference genome, Kaixuan 016.

High-quality SNPs were categorized based on the primary gene structure, including exonic regions, intronic regions, splicing sites, upstream and downstream regions, and intergenic regions. SNPs in coding exons were further dichotomized into those with synonymous mutations and those with nonsynonymous mutations, including those causing stop codons. PVE was analyzed using the Lm and ANOVA packages in the R language. Block analysis was conducted using the LDBlockShow 1.40 software [45]. The map of significant loci was drawn using the VennDiagram and UpSetR packages of the R language [46, 47]. Boxplots were drawn by R software. The significance of variation was evaluated using the t-test. KASP primers were synthesized by Golden Maker Technology (Beijing, China).

## Results

### Phenotypic diversity and heritability

The phenotypic observations of the nine traits under study were all continuous and followed a normal distribution. The mean values of the measured phenotypic traits, including HPW, HSW, SP, NP, NS, PL, PW, PC, and OC across four growth environments (Kaifeng in 2019, Xinyang in 2019, Kaifeng in 2020, Kaifeng in 2021) were 201.54 g, 73.07 g, 65.14%, 341.25, 449.22, 37.67 cm, 16.03 cm, 24.26%, and 50.05%, respectively (Table 1). The highest coefficients of variation (CV) were observed in NP and HPW, at 21.15% and 20.90%, respectively, while OC had the lowest CV at 3.97%. These results suggest that the environment had a greater impact on the phenotypes of NP and HPW than on those of OC. The broad-sense heritability (h^2^) of all nine traits ranged from 88.09 to 98.08%, indicating that the phenotypes of these traits were primarily determined by genetic factors (Table 1). Analysis of variance for nine traits indicated that the effect of genotypes, environments, and GE interactions were all significant (Table S1).

**Table 1:** Phenotypic variation of the nine traits in 199 accessions of four environments

Trait	Env	Max	Min	Mean	Variance	SD	CV (%)	Heritability (%)
HPW	E1	332.00	102.43	194.77	1371.02	37.03	19.01	97.06
	E2	351.30	123.13	213.20	1796.53	42.39	19.88	
	E3	303.68	109.83	196.33	1412.10	37.58	19.14	
	E4	355.25	105.90	200.21	1631.75	40.39	20.18	
HSW	E1	123.00	42.67	73.54	157.76	12.56	17.08	97.12
	E2	113.13	46.50	75.43	138.51	11.77	15.60	
	E3	108.67	45.40	70.15	113.73	10.66	15.20	
	E4	119.93	39.42	71.71	148.40	12.18	16.99	
SP	E1	75.73	54.41	67.35	17.70	4.21	6.25	95.16
	E2	74.63	46.80	63.88	35.48	5.96	9.32	
	E3	74.90	55.67	66.43	16.00	4.00	6.02	
	E4	72.37	49.24	62.27	24.83	4.98	8.00	
NP	E1	654.33	204.00	361.76	5004.59	70.74	19.56	96.87
	E2	540.00	176.21	330.33	4348.04	65.94	19.96	
	E3	526.25	193.71	332.45	3986.42	63.14	18.99	
	E4	577.28	203.40	334.00	3895.64	62.42	18.69	
NS	E1	749.67	248.67	446.34	5897.11	76.79	17.20	90.54
	E2	770.67	247.33	450.11	6764.22	82.24	18.27	
	E3	693.86	279.08	447.53	5252.76	72.48	16.19	
	E4	719.18	265.85	453.65	5714.52	75.59	16.66	
PL	E1	52.16	26.26	37.04	20.29	4.50	12.16	98.08
	E2	54.83	27.28	37.17	25.08	5.01	13.47	
	E3	52.36	27.72	38.26	21.65	4.65	12.16	
	E4	56.96	27.08	38.90	27.79	5.27	13.55	
PW	E1	19.84	11.21	15.98	2.35	1.53	9.59	96.45
	E2	20.39	11.68	16.44	2.63	1.62	9.87	
	E3	18.56	11.49	15.40	1.74	1.32	8.56	
	E4	20.79	12.06	16.13	2.54	1.60	9.89	
PC	E1	27.09	21.88	24.52	0.79	0.89	3.63	90.33
	E2	26.60	20.69	23.59	0.79	0.89	3.77	
	E3	28.99	23.25	26.31	1.14	1.07	4.06	
	E4	26.13	20.52	22.98	1.02	1.01	4.40	
OC	E1	53.59	46.14	49.91	1.30	1.14	2.29	88.09
	E2	54.63	48.19	51.48	1.44	1.20	2.34	
	E3	52.74	43.18	47.65	2.75	1.66	3.48	
	E4	54.18	47.16	50.49	1.46	1.21	2.39	

Note: HPW, hundred-pod weight; HSW, hundred-seed weight; SP, shelling percentage; NP, total number of 500 g of pods; NS, total number of 250 g of seeds; PL, pod length; PW, pod width; PC, protein content; OC, oil content; SD, standard deviation; CV, coefficients of variation; h^2^, broad-sense heritability; E1, Kaifeng in 2019; E2, Xinyang in 2019; E3, Kaifeng in 2020, E4, Kaifeng in 2021

**Fig. 1:**
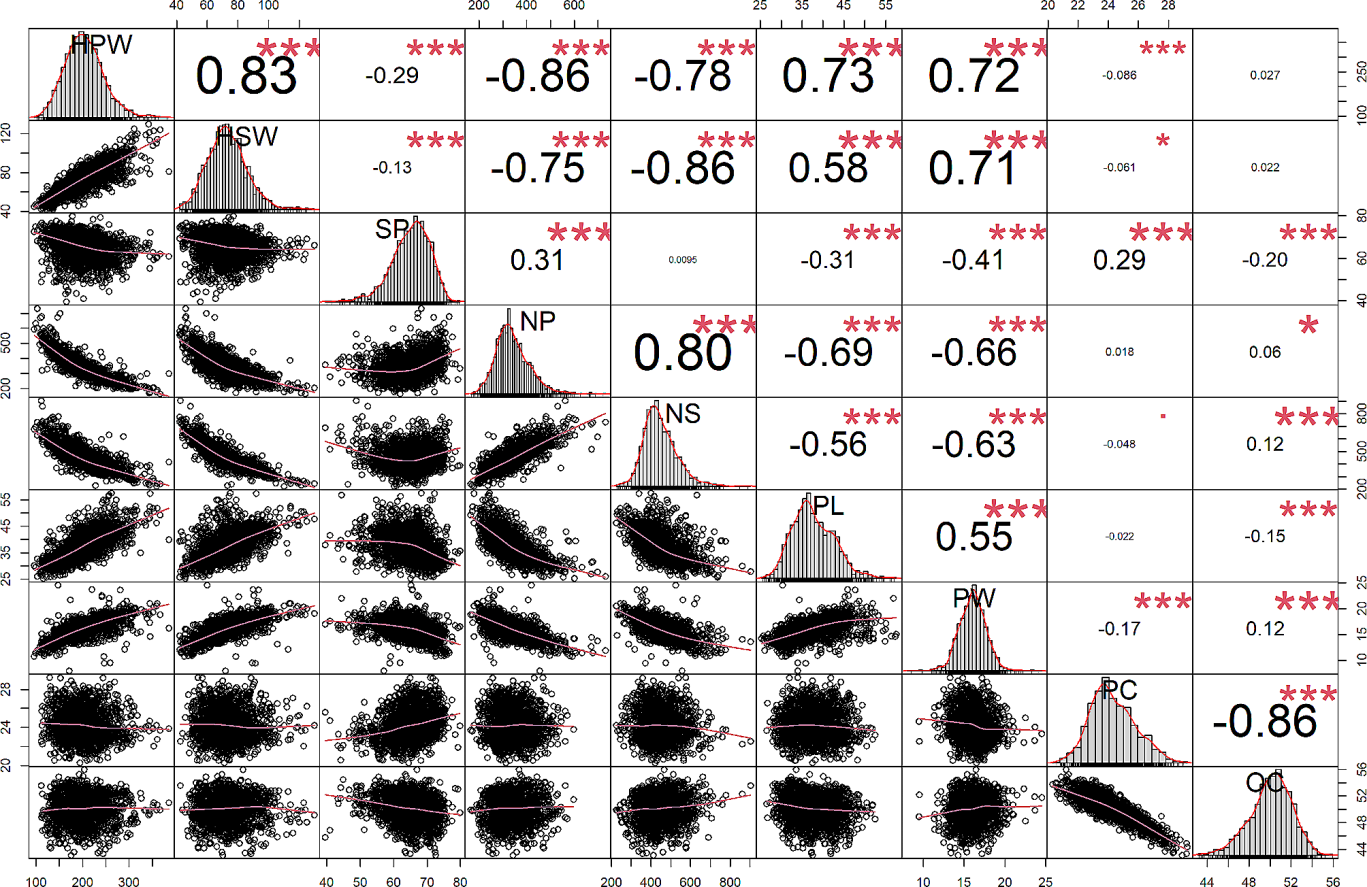
Correlation coefficient of the nine traits. HPW, hundred-pod weight; HSW, hundred-seed weight; SP, shelling percentage; NP, the total number of 500 g of pods; NS, the total number of 250 g of seeds; PL, pod length; PW, pod width. PC, protein content; OC, oil content. The number represents the correlation coefficient (r) value between traits. The circles in the lower half corner are distributed around the lines. r is high when the circles are located near the line. The greater the correlation between the traits, the larger the number in the upper left corner. ***, significant at *p* < 0.1% level, **, significant at *p* < 1% level, *, significant at *p* < 5% level

Among the seven traits of yield components, significant positive correlations were observed among HPW, HSW, PL, and PW, while significant negative correlations were found among SP, NP, and NS (Fig. 1). The highest positive correlation was between HPW and HSW, with a r value of 0.83, while the highest negative correlations were observed between HPW and NP, as well as between HSW and NS, both with a r value of -0.86. Regarding the quality traits, PC and OC exhibited a negative correlation, with a r value of -0.86. Except SP, NP, and NS, all other yield component traits exhibited negative correlations with PC and OC.

### Genomic variations of SNPs

**Fig. 2:**
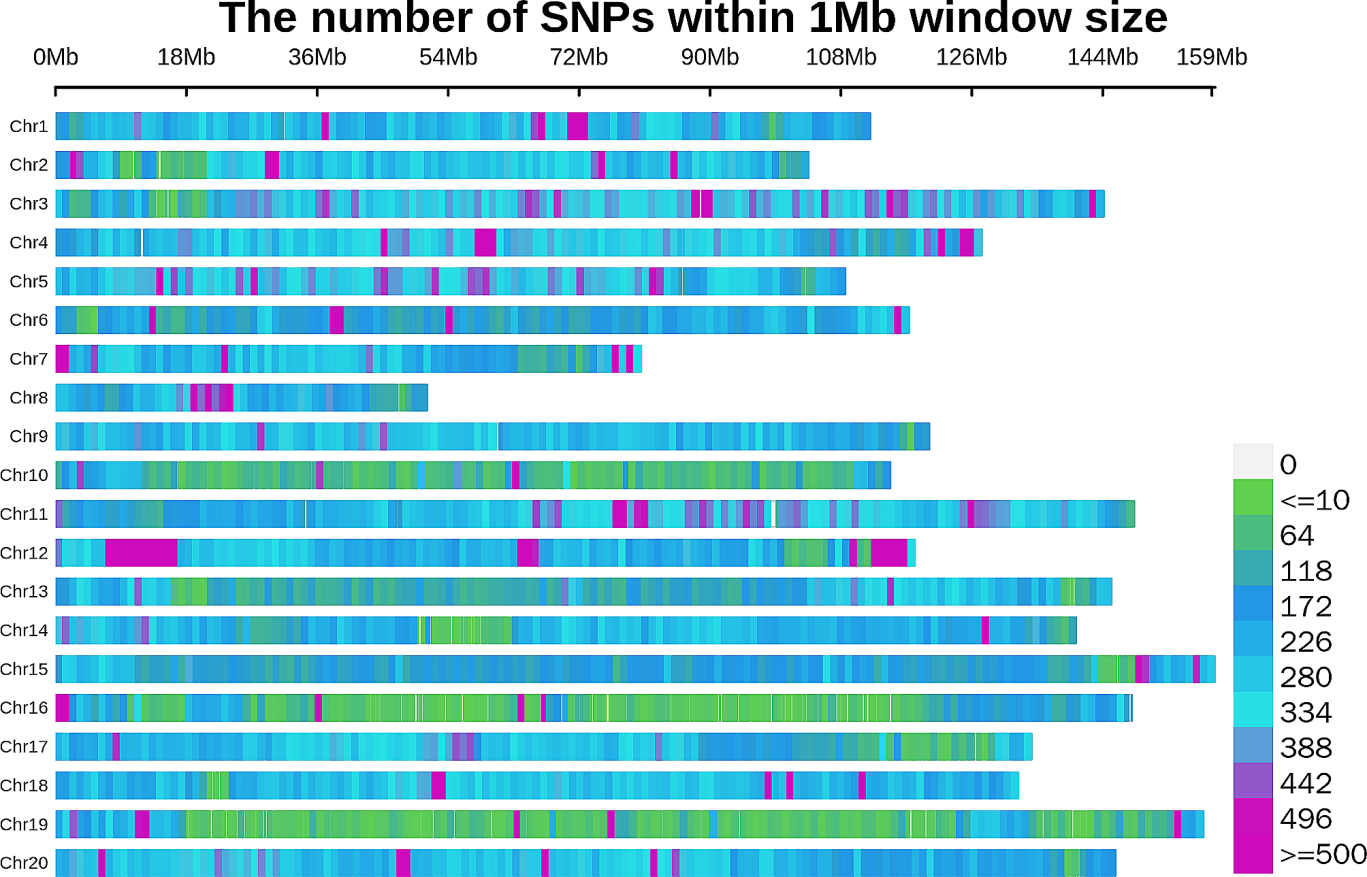
The density distribution of SNPs on peanut chromosomes. The ordinate represents the 20 chromosomes of Arachis hypogaea. The abscissa represents the length of the chromosomes. The color index represents the number of SNPs in 1.0 Mb window

The re-sequencing of all 199 peanut accessions with a sequencing depth of 10x generated a total of 7,056,911 Gb of raw data, with an average of 35,640,964 Mb of raw data per sample. The total amount of filtered, clean data was 7,048,906 Gb, with an average of 35,600,534 Mb per sample. After SNP calling and filtering, a total of 631,988 SNPs were retained. The highest number of SNPs was observed on Chr03, with 48,821, followed by Chr11 with 43,292 SNPs. In contrast, the lowest number of SNPs was found on Chr08, with 13,143 SNPs, followed by Chr10 with 13,848 SNPs. The average SNP density on chromosomes was 251.71/M (Fig. 2).

Functional annotation analysis revealed that 89.54% of the SNPs were situated in intergenic regions, with the remaining 10.46% identified within genic regions. Within these genic regions, a cumulative total of 26,899 SNPs were discernible in introns, subsequently followed by untranslated regions (UTRs) and coding regions of the annotated genes.

### Linkage disequilibrium decay

The LD decay was estimated by determining the LD coefficient (r^2^) between pairwise SNPs. Utilizing the parameters ‘-n -dprime-minMAF 0.05’ in PopLDdecay, the average r^2^ value was computed for pairwise markers within a 500 kb window and averaged across the entire genome. Following the suggestion of a previous study [48], the LD decay distance was defined as half of the maximum r^2^ value. Consequently, the peanut population’s LD was estimated to be 115 kb when r^2^ equaled 0.15 (Fig. S2).

### Population structure analysis

**Fig. 3:**
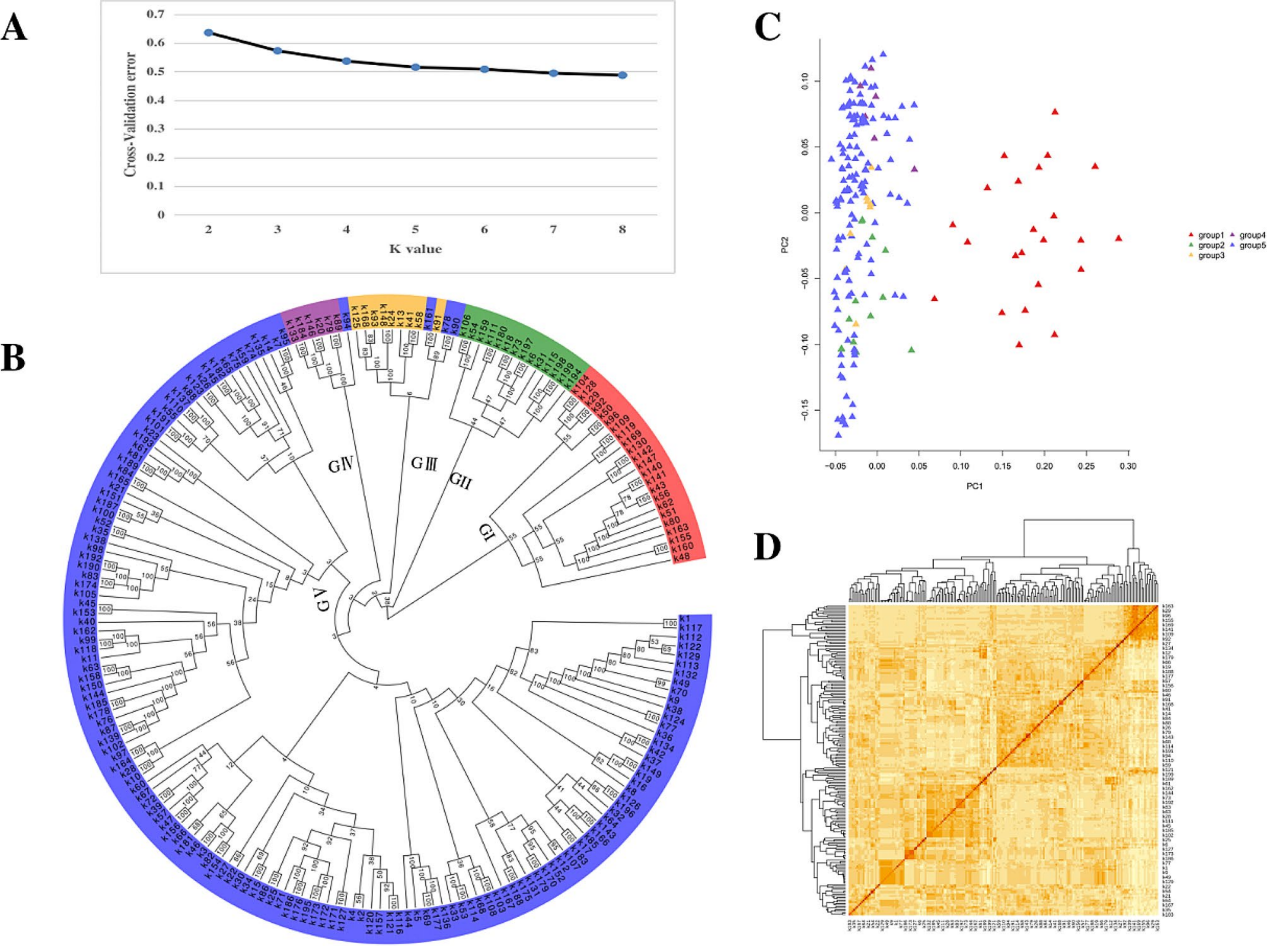
Genetic structures and phylogeny of the population. **A:** Population structure (k = 5). **B:** Phylogenetic tree. **C:** Principal component analysis (PCA). **D:** Heat map of pairwise relative kinship estimates

To understand the genetic structure of the population, 199 peanut accessions were categorized based on SNP data, and the class distribution of k values from 1 to 8 was calculated and simulated according to the Bayesian algorithm (Fig. 3A). When k equals five, the population can be segmented into five distinct groups. As shown in Fig. 3B, Group I (GI) comprised 23 accessions, all of which were multi-kernel types, resembling their paternal parent, Kainong 15. GroupII (GII) contained 14 accessions with PL to PW ratios less than 1.8. Group III (GIII) included 12 accessions, 75% of which exhibited a PL to PW ratio exceeding 2.7. Group IV (GIV) was consisted of seven varieties, six of them were derived from the same maternal parent. Group V (GV) was the largest category encompassing 143 accessions, all derived from advanced lines chosen during high-yield breeding (Fig. 3B; Dataset S1). PCA analysis based on the first (PC1) and the second (PC2) principal components revealed that, except for the first category, no distinct classification boundaries were evident (Fig. 3C). Similarly, the heat map generated using the k-matrix also displayed promiscuous boundaries between the five groups (Fig. 3D).

### Genome-wide association studies

**Fig. 4:**
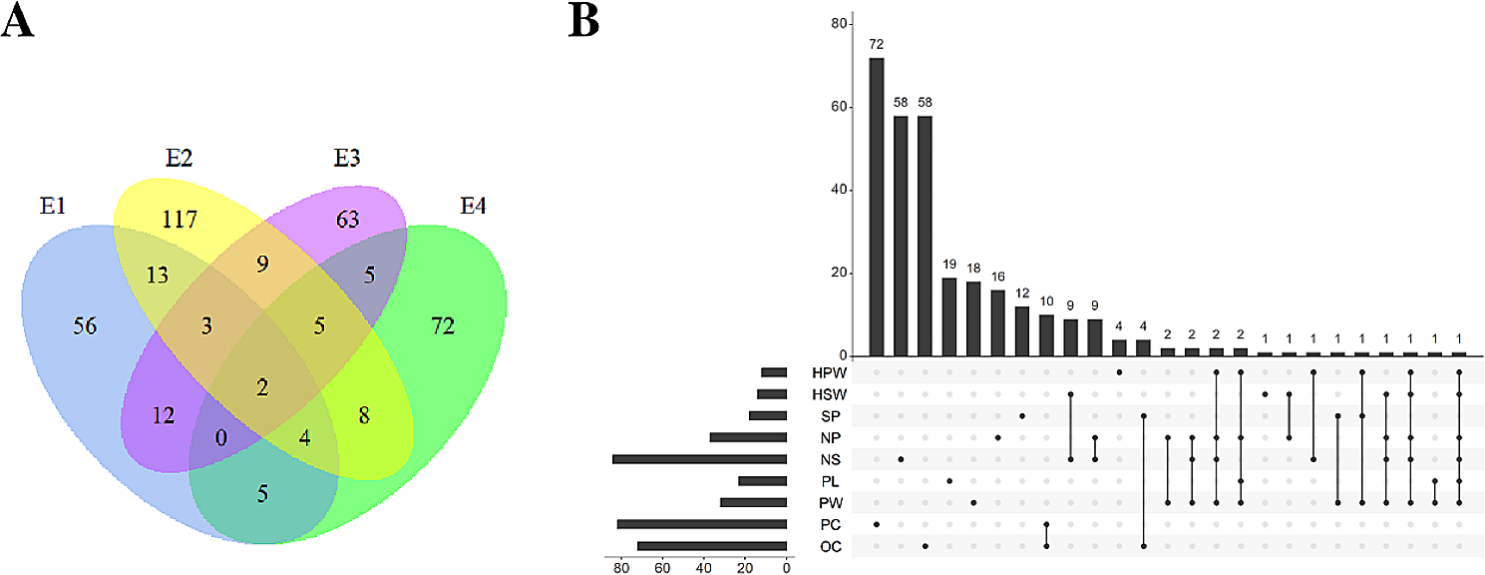
The profile of the associated loci for the selected nine traits of the 199 accessions across four different environments. **A:** The Venn diagram depicting the associated loci for the nine traits under four environments. **B:** The statistical column diagram representing the associations of the nine traits and their interactive upset plot. E1, Kaifeng in 2019; E2, Xinyang in 2019; E3, Kaifeng in 2020; E4, Kaifeng in 2021; HPW, hundred-pod weight; HSW, hundred-seed weight; SP, shelling percentage; NP, the total number of 500 g of pods; NS, the total number of 250 g of seeds; PL, pod length; PW, pod width

In the association panel comprising 199 peanut accessions, a total of 631,988 SNPs (minor allele frequency ≥ 0.05; missing rate ≤ 0.2; depth ≥ 3) were used for the GWAS analysis. The threshold value of GWAS, determined by the Bonferroni test, was 7.10 [- log10 (0.05/631,988)]. A total of 95, 161, 99, and 101 maker traits associations (MATs) were identified in the environments E1, E2, E3, and E4, respectively (Fig. 4A; Table S2). The number of SNPs detected for individual traits varied across different environments. In E3, the maximum number of SNPs detected for HPW and SP was 7 and 14, respectively. In E2, the maximum number of SNPs detected for HSW, NP, PL, and PC was 11, 17, 17, and 39, respectively. The highest numbers of SNPs for NS and PW were detected in E4 and E1, respectively. No SNP was discernible for HPW and HSW in E4, nor for SP in E2. A total of 12, 14, 18, 37, 84, 23, 32, 82, and 72 non-redundant association sites were identified for HPW, HSW, SP, NP, NS, PL, PW, PC, and OC, respectively (Fig. 4B; Table S2). The associated loci of yield components were primarily situated on Chr08 and Chr16, with a lesser extent on Chr13, while those of the two quality traits were predominantly located on Chr08 and Chr18. The Manhattan plots and quantile-quantile plots of each trait under the four environments are shown in Fig. S3.

A total of 374 significant loci were detected for the nine traits, with 66 of these detected in more than two environments. For example, Arahy.16_142867474 for PL and Arahy.16_142692237 for PW were detected in all four environments (Fig. 4A). Forty-eight SNP loci were attributable to two or more traits. For instance, Arahy.16_142692237, Arahy.16_139632313, Arahy.16_142656321, and Arahy.16_138643609 were associated with six, five, four, and three yield components, respectively. The repeatedly detected loci for quality traits were all located on Chr08, while those related to yield traits were primarily found on Chr16. Apart from SP, no SNP overlap was observed between quality traits and yield traits (Fig. 4B; Table S3).

### Verification of the relationship between SNPs and phenotype

To verify the associated loci of the selected seven yield traits and two quality traits in peanuts, we extracted the genotypes of seven SNP loci from 199 accessions and chose approximately 50 extreme phenotypes for each trait to construct box plots using the following criteria: HPW ≥ 220 g, HPW ≤ 160 g, HSW ≥ 80 g, HSW ≤ 65 g, SP ≥ 69%, SP ≤ 62%; NP ≥ 380, NP ≤ 290, NS ≥ 500, NS ≤ 390, PL ≥ 41 cm, PL ≤ 34 cm, PW ≥ 17 cm, PW ≤ 15 cm, PC ≥ 25%, PC ≤ 23.6%, OC ≥ 50%, OC ≤ 49%. Significant phenotypic differences between the two base types for the nine traits were evident (Dataset S2). At the Arahy16_138643609 site, the high-yield genotypes had a CC base, in contrast to TT in the low yield genotypes. For Arahy.16_139632313 and Arahy.16_142692237, the high-yield genotypes had a GG base, while the low-yield genotypes had an AA base. In Arahy.16_142656321, the high-yield genotypes featured a TT base, as opposed to CC in the low-yield genotypes. In Arahy.16_142867474, the high-yield genotypes had a GG base, while the low-yield genotypes had a CC base. At Arahy.08_38378278, the high-oil and low-oil genotypes had TT and CC bases, respectively. In Arahy.08_49538603, the high-PC/low-OC genotypes had an AA base, in contrast to CC in the low-PC/high-OC genotypes (Fig. S4). The genotype proportion of CC/TT, GG/AA, GG/AA, TT/CC, GG/CC, TT/CC and CC/AA were 168/25, 150/27, 158/28, 156/27, 157/33, 116/41 and 136/45, respectively (Dataset S2).

### Candidate genomic regions for yield and quality traits

**Fig. 5:**
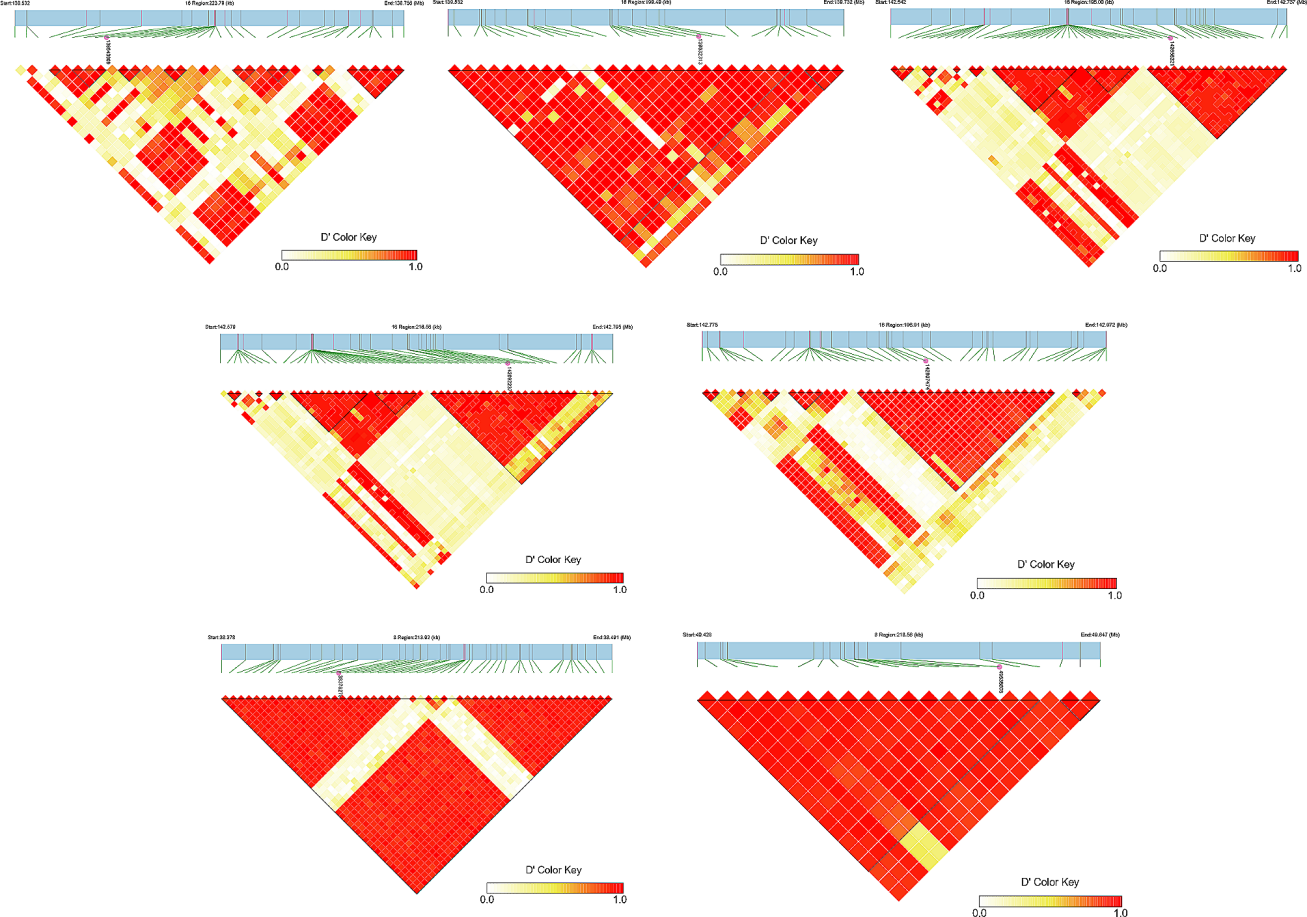
LD block surrounding the significant SNPs. The pairwise Linkage Disequilibrium (LD) between the SNP markers is indicated as D’ values, where red indicates a value of 1 and yellow indicates 0. Dark red represents the highest correlation and the highest LD between the two SNPs. D’ is the standardized linkage disequilibrium coefficient

Yield trait loci were predominantly clustered on Chr16, where SNP Arahy.16_138643609, Arahy.16_42656321, and Arahy.16_142867474 were repeatedly identified in association with four yield traits across multiple environments. Additionally, SNP Arahy.16_139632313 and Arahy.16_142692237 were associated with five and six yield traits, respectively, in multiple environments. The highest PVE of yield component trait loci in different environments ranged from 17.64 to 32.61% (Table 2). Arahy.16_139632313, which was closely related to five yield traits, accounting for 26.43% of the phenotypic variation of HPW in E1. Arahy.16_142692237 and Arahy.16_142867474 were consistently found in association with PL and PW in all four environments. Arahy.16_142692237 and Arahy.16_142867474 accounted for 17.64% and 21.17% of the phenotypic variations in E3 and E2, respectively. In contrast, Arahy.08_38378278 and Arahy.08_49538603 were linked with quality traits, accounting for 12.86% and 14.06% of the phenotypic variation of OC in E1 and E3, respectively. Analysis of the 115 kb region upstream and downstream of these sites using the LD haplotype block diagram revealed that the seven significant SNP sites formed seven blocks, including a total of 158 SNPs (Fig. 5). Among these seven blocks, the peak value (- log10P) of yield traits ranged from 7.11 to 10.48 in different environments, and the peak value of quality traits ranged from 5.06 to 7.06 (Table S4).

**Table 2:** The repeatedly detected major association loci of the nine yield components and quality traits in peanuts

SNP	Ref	Alt	Trait (No. of environment detected the signal)	Max PVE (%)
Arahy.16_138643609	C	T	HSW(2), NP(2), NS(2), PW(2)	19.79
Arahy.16_139632313	G	A	HPW(1), HSW(2), NP(2), NS(1), PW(3)	26.43
Arahy.16_142656321	C	T	HPW(2), NP(3), NS(1), PW(2)	32.61
Arahy.16_142692237	G	A	HPW(2), HSW(2), NP(2), NS(2), PL(1), PW(4)	17.64
Arahy.16_142867474	C	G	HPW(1), NP(1), PL(4), PW(3)	21.17
Arahy.08_38378278	C	T	PC(1), OC(1)	12.86
Arahy.08_49538603	A	T	PC(2), OC(1)	14.06

HPW, hundred-pod weight; HSW, hundred-seed weight; SP, shelling percentage; NP, total number of 500 g of pods; NS, total number of 250 g of seeds; PL, pod length; PW, pod width; PC, protein content; OC, oil content. PVE, phenotypic variation explained

Using Kaixuan 016 as the reference genome, we discovered that five SNPs located in the coding regions of several key genes resulted in nonsynonymous variations (Table 3). Gene function annotation analysis revealed that one of the SNPs, Arahy.16_142682809, was situated within the coding region of gene evm.TU.ctg197.486, which encodes a pyruvate monooxygenase. This SNP led to an amino acid substitution of Lys to Asn in the fourth exon, resulting from a nucleotide substitution of A to C. Another SNP, Arahy.16_142783542, was identified in the coding region of evm.TU.ctg197.494, which encodes a shikimate_O-hydroxycinnamoyltransferase. The SNP caused an amino acid alteration from Arg to Lys. Moreover, the Arahy.08_38352339 SNP was discovered in the second exon of evm.TU.ctg335.1373, encoding an NAC transcription factor. A nucleotide change from C to A led to the formation of a stop codon. The Arahy.08_38476979 SNP was found in the third exon of evm.TU.ctg335.1377, encoding an NAC domain-containing protein. A nucleotide alteration from A to G resulted in an amino acid substitution from Ile to Val. Finally, the Arahy.08_49440731 SNP was located in the first exon of evm.TU.ctg335.2426, which encodes a protein predicted to be involved in carbohydrate metabolism. This SNP caused a Thr-to-Ile amino acid substitution due to a C-to-T nucleotide alteration.

**Table 3:** The non-synonymous mutation loci annotation in the genomic regions

Linkage SNP loci	Gene name	Gene start	Gene end	Gene length	Ref	Alt	Protein substitution	Functional annotation
Arahy.16_142682809	evm.TU.ctg197.486	142,682,800	142,685,882	3083	A	C	Lys-Asn	Pyruvate monooxygenase
Arahy.16_142783542	evm.TU.ctg197.494	142,781,095	142,783,744	2650	G	A	Arg-Lys	Shikimate O-hydroxycinnamoyltransferase
Arahy.08_38352339	evm.TU.ctg335.1373	38,351,075	38,352,916	1842	C	A	Tyr-Stopgain	NAC transcription factor
Arahy.08_38476979	evm.TU.ctg335.1377	38,475,835	38,477,774	1940	A	G	Ile-Val	NAC domain-containing protein
Arahy.08_49440731	evm.TU.ctg335.2426	49,439,949	49,441,823	1875	C	T	Thr-Ile	Carbohydrate metabolic

### KASP markers validation of the SNPs

The association between the five aforementioned SNPs and peanut yield components and quality traits was validated by analyzing re-sequencing data and evaluating the phenotypic performance of the population. At the Arahy16_142682809 site, the GG genotype was associated with high yield, whereas the TT genotype was linked to low yield. Similarly, the high-oil trait was associated with the GG genotype at the Arahy.08_38352339 site. For the Arahy.08_49440731 site, the GG genotype was associated with high-PC/low-OC, while the low-PC/high-OC feature was associated with the AA genotype (Fig. S5). However, no variation was observed among the population at the Arahy.16_142783542 site and the SNP in Arahy.08_38476979 did not lead to any significant phenotypic variation.

**Fig. 6:**
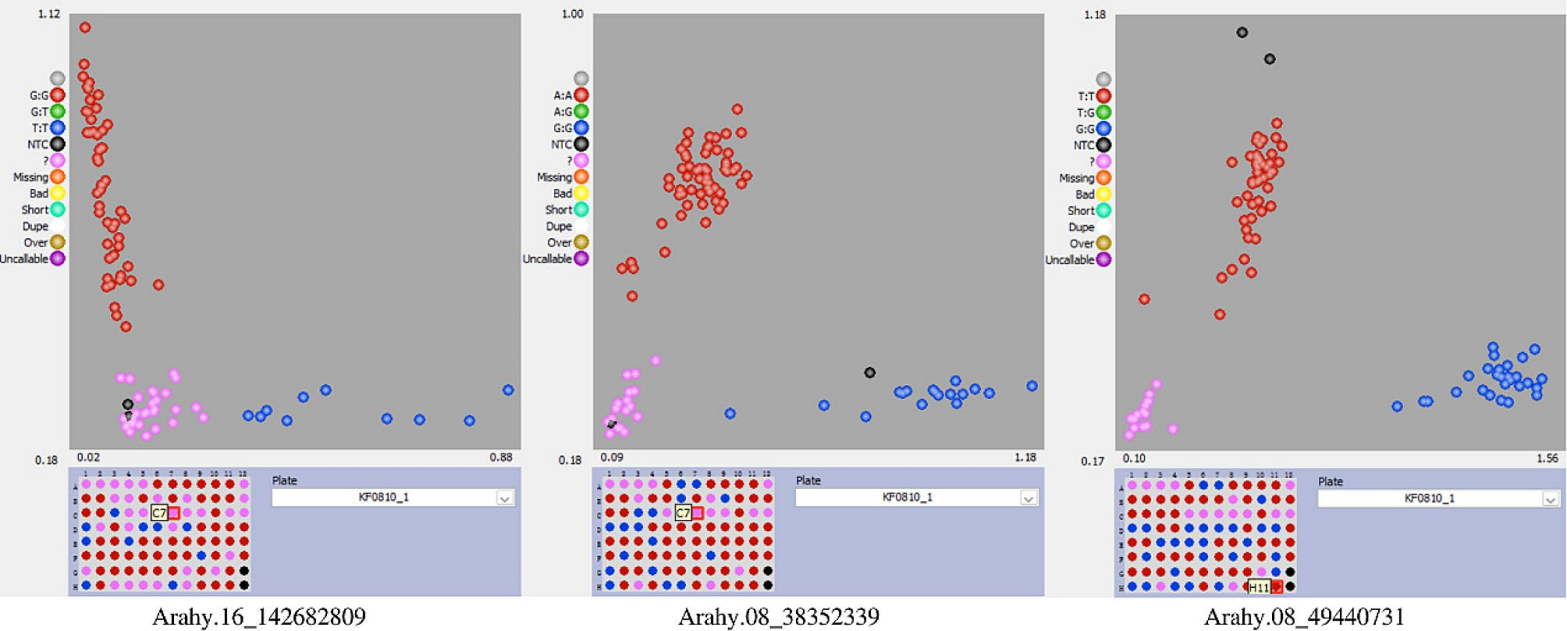
Kompetitive allele-specific PCR (KASP) markers for the validation of three selected SNPs, Arahy16_142682809, Arahy.08_38352339, Arahy.08_49440731

**Fig. 7:**
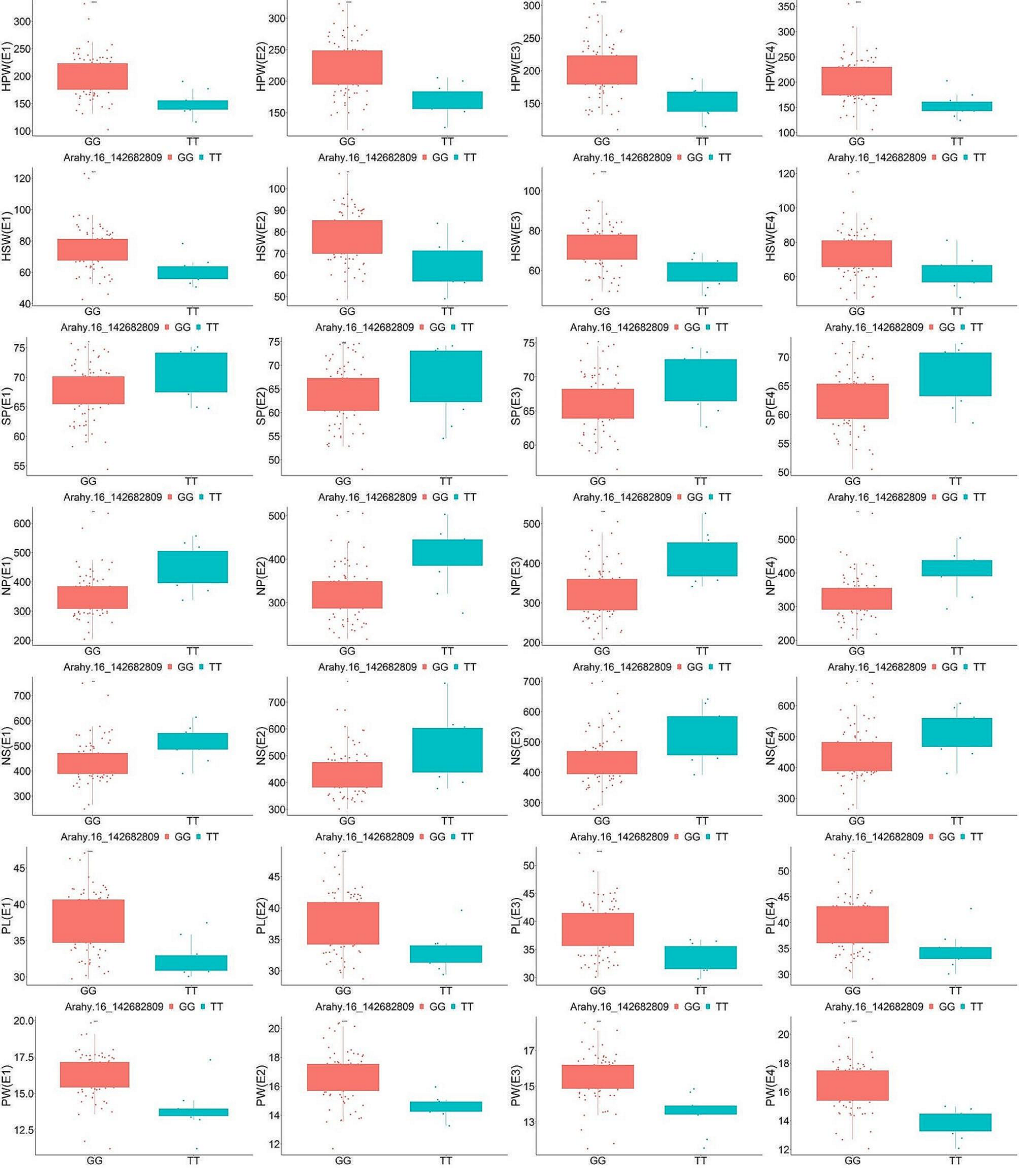
Kompetitive allele-specific PCR (KASP) validation of the phenotypic variations between two base types at Arahy.16_142682809. E1, Kaifeng in 2019; E2, Xinyang in 2019; E3, Kaifeng in 2020; E4, Kaifeng in 2021; HPW, hundred-pod weight; HSW, hundred-seed weight; SP, shelling percentage; NP, total number of 500 g of pods; NS, total number of 250 g of seeds; PL, pod length; PW, pod width

**Fig. 8:**
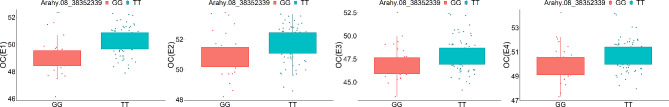
Kompetitive allele-specific PCR (KASP) validation of the phenotypic variation between two base types at Arahy.08_38352339. E1, Kaifeng in 2019; E2, Xinyang in 2019; E3, Kaifeng in 2020; E4, Kaifeng in 2021; OC, oil content

**Fig. 9:**
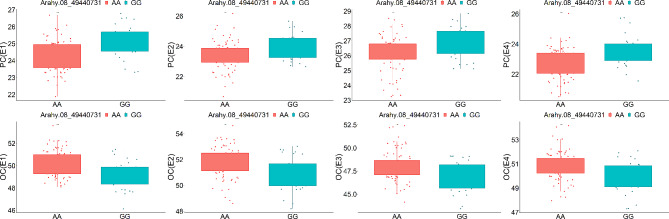
Kompetitive allele-specific PCR (KASP) validation of the phenotypic variation between two base types at Arahy.08_49440731. E1, Kaifeng in 2019; E2, Xinyang in 2019; E3, Kaifeng in 2020; E4, Kaifeng in 2021; PC, protein content; OC, oil content

Subsequently, KASP markers were designed for Arahy.16_142682809, Arahy.08_38352339, and Arahy.08_49440731 and validated in a total of 199 peanut accessions (Table S5, Fig. 6). The results, illustrated in Figs. 7 and 8, and 9, demonstrated that these KASP markers can effectively differentiate the SNPs at Arahy.16_142682809, Arahy.08_38352339, and Arahy.08_49440731 with clarity and precision.

## Discussion

Peanut yield and quality are crucial for the thriving peanut industry, as they directly impact its economic value and global competitiveness. GWAS has emerged as a powerful tool for identifying key genes that influence these essential traits. By unraveling the genetic architecture of high-yield and superior-quality peanuts, GWAS enables the development of marker-assisted breeding programs. This targeted approach accelerates the breeding process, ensuring the cultivation of high-performing peanut varieties that cater to consumer demand and market needs. In turn, this advances the industry’s sustainability and profitability, benefiting both producers and consumers alike.

This study analyzed 199 peanut accessions, all originating from the high-oleic acid-containing Chinese variety Kaixuan 016 [49, 50]. Genotyping analysis of this diverse population, featuring significant variation in yield components and quality parameters, was enabled by using Kaixuan 016 as a reference genome. The presence of a normal distribution of phenotypic data (Fig. 1), coupled with pedigree analysis and the genetic contribution of Kaixuan 016 to derived lines (as per unpublished data), indicated a rich genetic diversity among the 199 accessions.

The substantial phenotypic diversity displayed across four distinct growth environments enhances genotyping efficiency and improves the resolution of GWAS analyses. By monitoring the population for LD decay, we made accurate estimates regarding the required number and granularity of markers for meaningful association analysis [51–53]. Given Kaixuan 016’s reference genome size of 2.53 Gb, we estimated that 22,528 SNP markers (genome size/LD attenuation distance) would be necessary for accurate association analysis. However, 631,988 SNPs were identified in this study, representing the most abundant high-quality SNP loci obtained via the 10× deep sequencing of peanuts to date, far exceeding the number used in prior studies [17, 54, 55].

It was observed that the CV values for yield traits were higher than those for quality traits across all four environments, aligning with other studies that found yield traits to be more susceptible to environmental influence [56, 57]. Broad-sense heritability for all nine traits exceeded 80%, indicating that phenotypes were primarily determined by genotype [58]. A quantitative trait like yield can have a high heritability as the experiment was well conducted with high appropriate replication levels, and this higher heritability had also been observed in other studies [59, 60]. Among yield traits, HPW, HSW, PL, and PW were positively correlated with one another, while SP displayed a significant negative correlation. Larger pods were associated with less plump kernels, likely due to homeostatic compensatory effects influencing plant growth and development. Furthermore, a negative correlation was observed between PC and OC, consistent with the competitive metabolism and accumulation of oil and proteins as major storage compounds in peanut seeds [27].

Investigating a population’s genetic structure can clarify its origin, composition, and evolution, while controlling false positives produced by population structure through PCA and structural analysis. The 199 accessions were categorized into five groups in this study. Kainong 15, the paternal parent of all 23 accessions in GI, is a red testa variety with 3–4 kernels per pod. The heritability of multi-kernel pods was strong, with all progenies derived from Kainong 15 being multi-kernelled. Peanut populations GII, GIII, and GV represent small, large, and medium-sized pods, respectively. This variation in pod size between groups enables the easier identification of significant linkage loci for economic traits through association analysis.

In this study, GWAS analysis identified 374 significant SNP loci associated with nine traits, including 220 for yield traits and 154 for quality traits (Fig. 4B; Table S2). The number of associated loci identified substantially exceeded those reported in previous studies [10, 11, 17, 25, 57, 61]. We pinpointed several candidate genes, such as those encoding pyruvate monooxygenase, shikimate_O-hydroxycinnamoyltransferase, and the NAC transcription factor, which are potentially associated with yield component and quality traits. Pyruvate monooxygenases catalyze the conversion of pyruvate to acetyl-CoA, a central molecule in cellular metabolism, including the citric acid cycle and fatty acid biosynthesis [62]. Shikimate_O-hydroxycinnamoyltransferase is involved in the phenylpropeanoid biosynthesis pathway and plays a crucial role in synthesizing various secondary metabolites such as lignins, flavonoids, and phenolic acids [63]. These compounds contribute to the seed coat’s structure and integrity, which are vital for protecting the developing embryos and maintaining seed viability. Although not directly associated with seed storage compound accumulation, its involvement in the phenylpropanoid pathway can have indirect effects on these processes [64, 65].

NAC transcription factors are plant-specific proteins with essential regulatory roles in plant development and stress responses. In peanut kernel development, NAC transcription factors may regulate gene expression related to cell differentiation, expansion, and maturation, as well as the response to environmental stress factors impacting flower and kernel development [66]. Overexpression of *OsNAC6* in rice leaded to growth hindrance and yield reduction [67]. Additionally, grape *NAC26* polymorphism had been linked to fruit size variations [68]. Given that these genes may play a role in providing the necessary energy and precursors for the synthesis of lipids, proteins, and other biomolecules during seed development, it is plausible to assume that the SNPs present in these genes may affect their function and impact peanut kernel yield or quality in one way or another. Three candidate genes associated with peanut yield and quality traits, specifically evm.TU.ctg197.486, evm.TU.ctg335.1373, and evm.TU.ctg335.2426 were identified to be *arahy.6PM354, arahy.65HUV4*, and *arahy.QNIR3T* respectively in Tifrunner genome (https://www.peanutbase.org/peanut_genome). Among them, the oil content candidate gene *arahy.65HUV4* was reported in the previous study [69]. A BLAST search within NCBI (National Center for Biotechnology Information) databases revealed that the other two genes were not reported.

Three KASP markers were developed for SNPs at Arahy.16_142682809 (yield component traits) and Arahy.08_38352339 and Arahy.08_49440731 (quality traits). The effectiveness of these markers was verified, suggesting their potential as selection markers for yield and quality traits. These markers could prove valuable for the fine mapping of candidate genes and can be directly applied in peanut breeding programs to enable accurate and effective selection of desired traits. However, further research is necessary to validate the function of these SNPs and comprehend the underlying biological mechanisms involved. Acquiring this knowledge is crucial for optimizing the accuracy and efficiency of marker-assisted breeding as well as recognizing any limitations or constraints that may arise when implementing these markers in breeding programs.

### Electronic supplementary material

Below is the link to the electronic supplementary material.

Supplementary Material 1

Supplementary Material 2

Supplementary Material 3

Supplementary Material 4

Supplementary Material 5

Supplementary Material 6

Supplementary Material 7

Supplementary Material 8

Supplementary Material 9

Supplementary Material 10

Supplementary Material 11

Supplementary Material 12

## Data Availability

The re-sequencing datasets of the 199 peanut materials have been deposited in the NCBI Sequence Read Archive (SRA) under accession PRJNA974180 (https://www.ncbi.nlm.nih.gov/sra/PRJNA974180).

## Abbreviations

GWAS: Genome-wide association study
SNP: Single nucleotide polymorphism
LD: Linkage disequilibrium
KASP: Kompetitive allele-specific PCR
InDels: Insertions and deletions
NJ: Neighbor-joining
UTR: Untranslated region
CV: Correlation of variation
HPW: Hundred-pod weight
HSW: Hundred-seed weight
SP: Shelling percentage
NP: Total number of 500 g of pods
NS: Total number of 250 g of seeds
PL: Pod length
PW: Pod width
PC: Protein content
OC: Oil content
