# Supplementary material for: Genome-wide association study and development of molecular markers for yield and quality traits in peanut (Arachis hypogaea L.)
Source: BMC Plant Biol. 2024 Apr 5;24:244. doi: 10.1186/s12870-024-04937-5 (PMC10996145; doi:10.1186/s12870-024-04937-5)
Supplement: Supplementary file 1 — Supplementary Material 1 [file 12870_2024_4937_MOESM1_ESM.pdf]

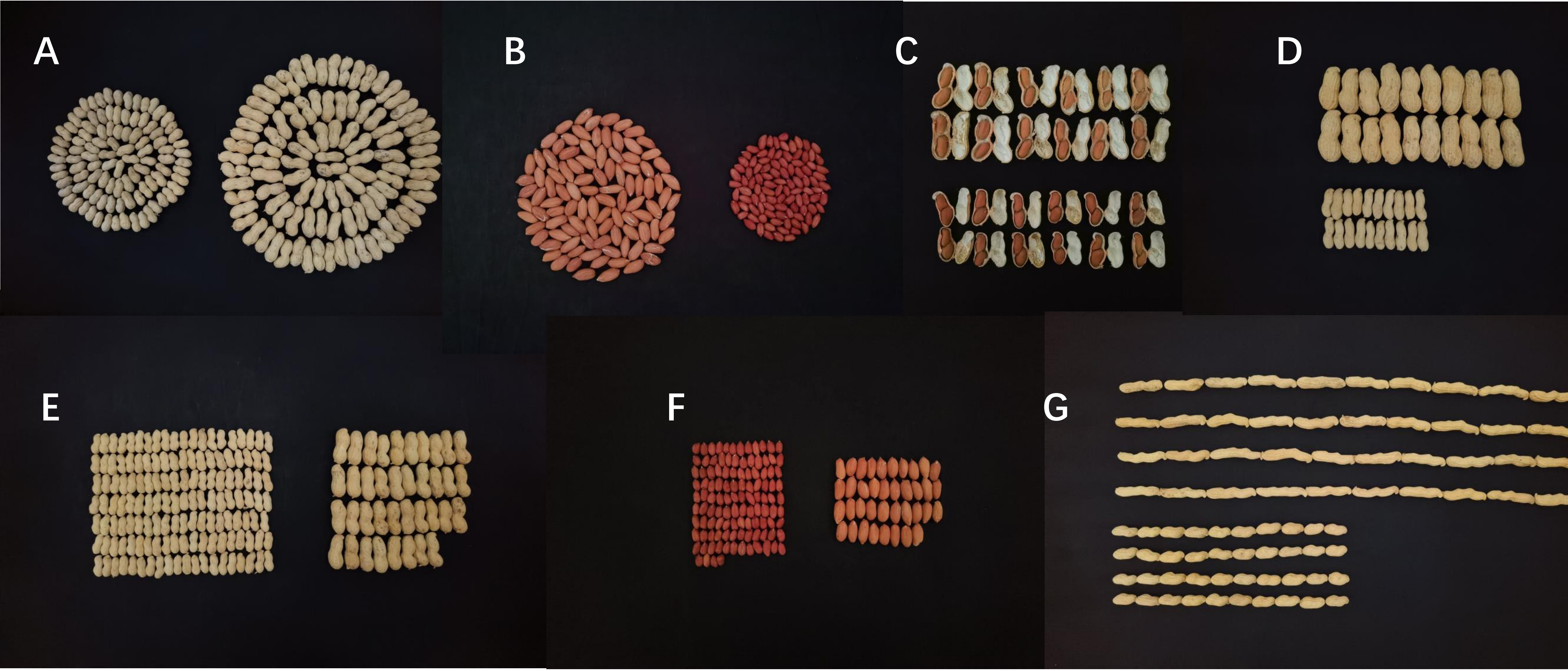

Fig. S1. Contrary phenotypes of the seven yield traits. A-G: Contrary phenotypes of hundred-pod weight, hundred-seed weight, shelling percentage, pod width, No. of pods per 200 g pods, No. of seeds per 50 g seeds, and pod length.
