# Supplementary material for: Genome-wide association study and development of molecular markers for yield and quality traits in peanut (Arachis hypogaea L.)
Source: BMC Plant Biol. 2024 Apr 5;24:244. doi: 10.1186/s12870-024-04937-5 (PMC10996145; doi:10.1186/s12870-024-04937-5)
Supplement: Supplementary file 2 — Supplementary Material 2 [file 12870_2024_4937_MOESM2_ESM.pdf]

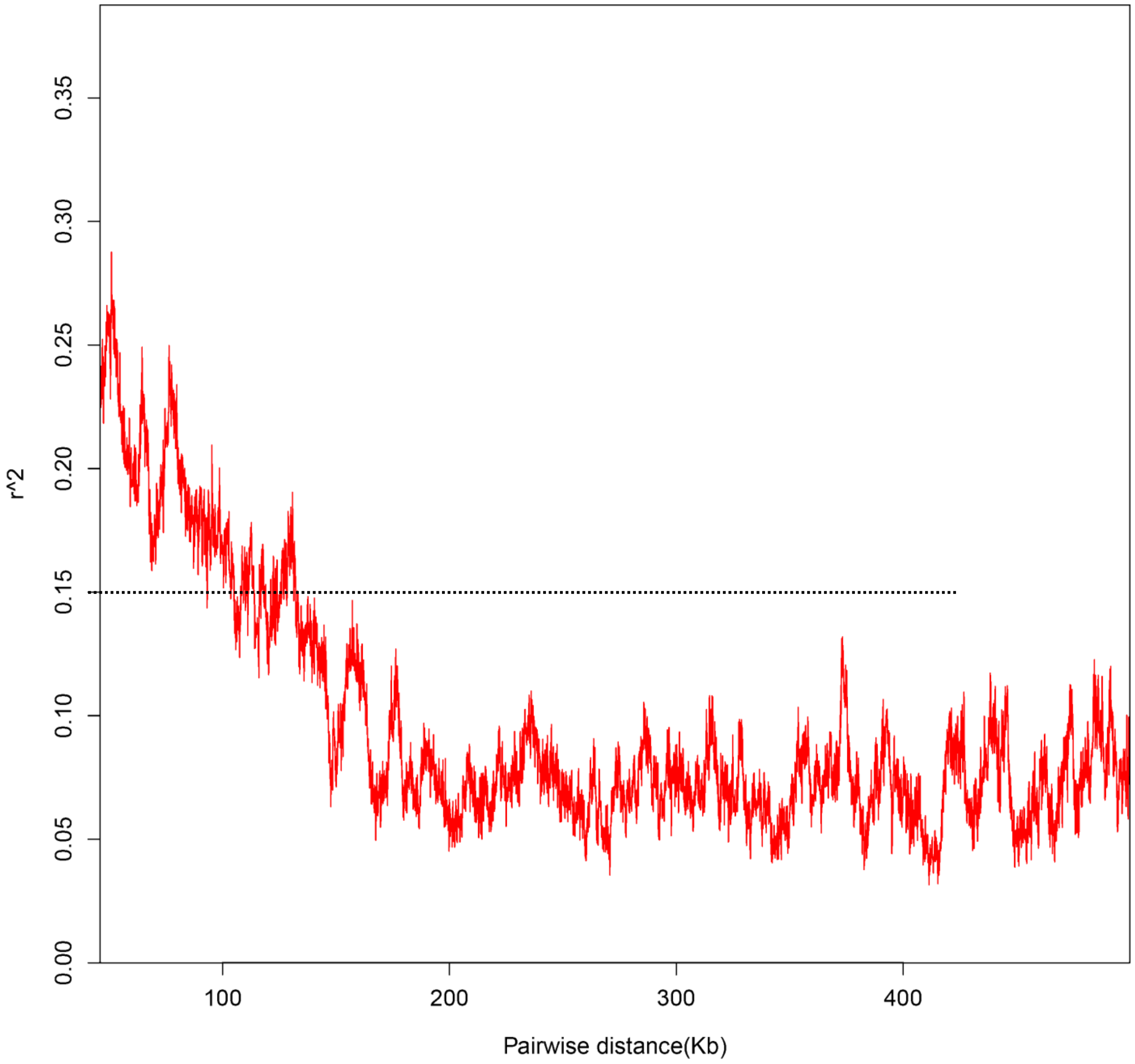

Fig. S2. Linkage disequilibrium (LD) decay for the 199 Chinese peanut accessions. r^2 is the LD coefficient.
