# Supplementary material for: Genome-wide association study and development of molecular markers for yield and quality traits in peanut (Arachis hypogaea L.)
Source: BMC Plant Biol. 2024 Apr 5;24:244. doi: 10.1186/s12870-024-04937-5 (PMC10996145; doi:10.1186/s12870-024-04937-5)
Supplement: Supplementary file 3 — Supplementary Material 3 [file 12870_2024_4937_MOESM3_ESM.pdf]

A

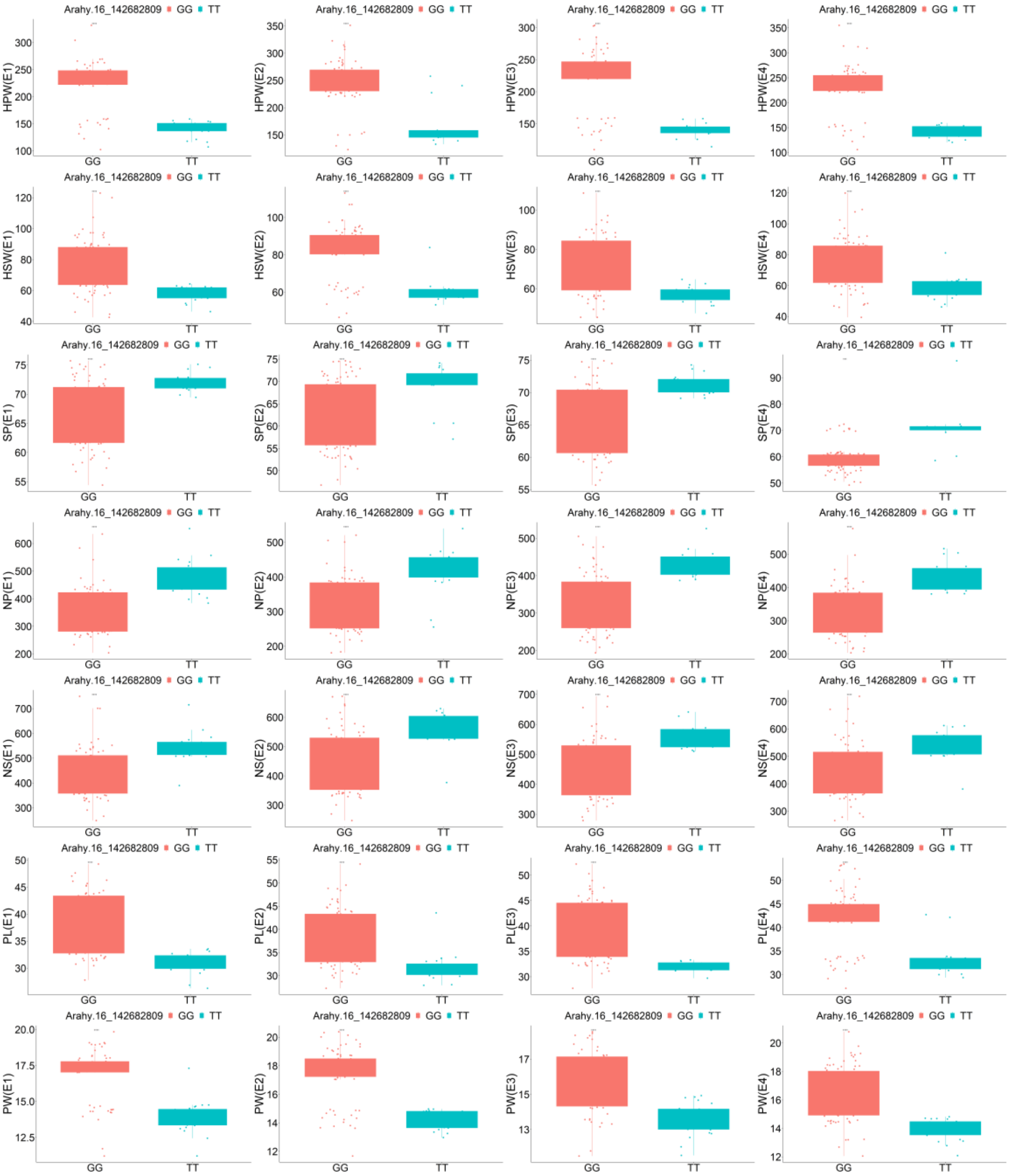
Arahy.16_142682809 GG TT
HPW(E1)
HPW(E2)
HPW(E3)
HPW(E4)
HSW(E1)
HSW(E2)
HSW(E3)
HSW(E4)
SP(E1)
SP(E2)
SP(E3)
SP(E4)
NP(E1)
NP(E2)
NP(E3)
NP(E4)
NS(E1)
NS(E2)
NS(E3)
NS(E4)
PL(E1)
PL(E2)
PL(E3)
PL(E4)
PW(E1)
PW(E2)
PW(E3)
PW(E4)
GG
TT

B

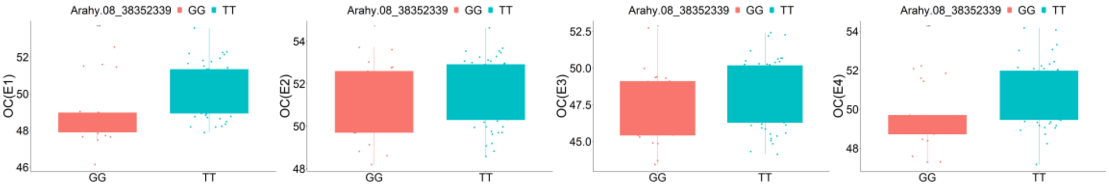
Arahy.08_38352339 GG TT
OC(E1)
OC(E2)
OC(E3)
OC(E4)
GG
TT

C

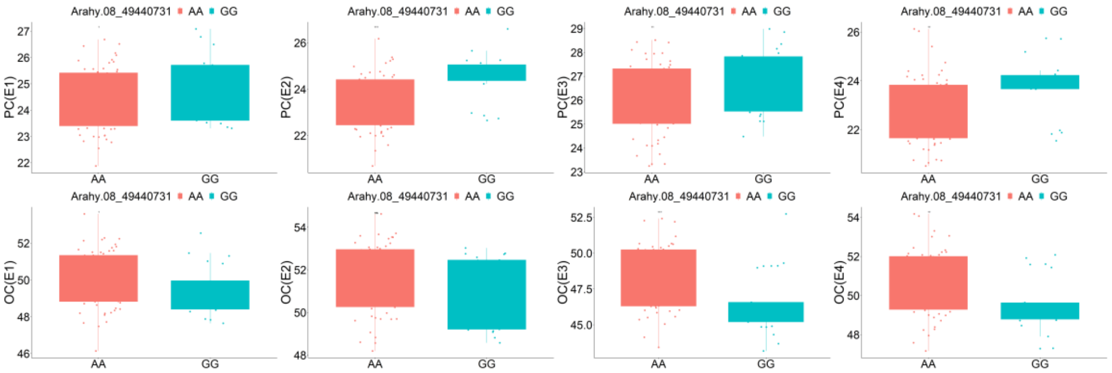

Fig. S5. Phenotypic difference between two base types at three nonsynonymous SNPs. A-C: Phenotypic difference between two base types at Arahy16_142682809, Arahy.08_38352339, and Arahy.08_49440731. HPW, hundred-pod weight; HSW, hundred-seed weight; NP, total number of 500 grams of pods; NS, total number of 250 grams of seeds; PL, pod length; PW, pod width. PC, protein content; OC, oil content. E1, Kaifeng in 2019; E2, Xinyang in 2019; E3, Kaifeng in 2020; E4, Kaifeng in 2021.
