# Supplementary material for: Genome-wide association study and development of molecular markers for yield and quality traits in peanut (Arachis hypogaea L.)
Source: BMC Plant Biol. 2024 Apr 5;24:244. doi: 10.1186/s12870-024-04937-5 (PMC10996145; doi:10.1186/s12870-024-04937-5)
Supplement: Supplementary file 4 — Supplementary Material 4 [file 12870_2024_4937_MOESM4_ESM.pdf]

**A**

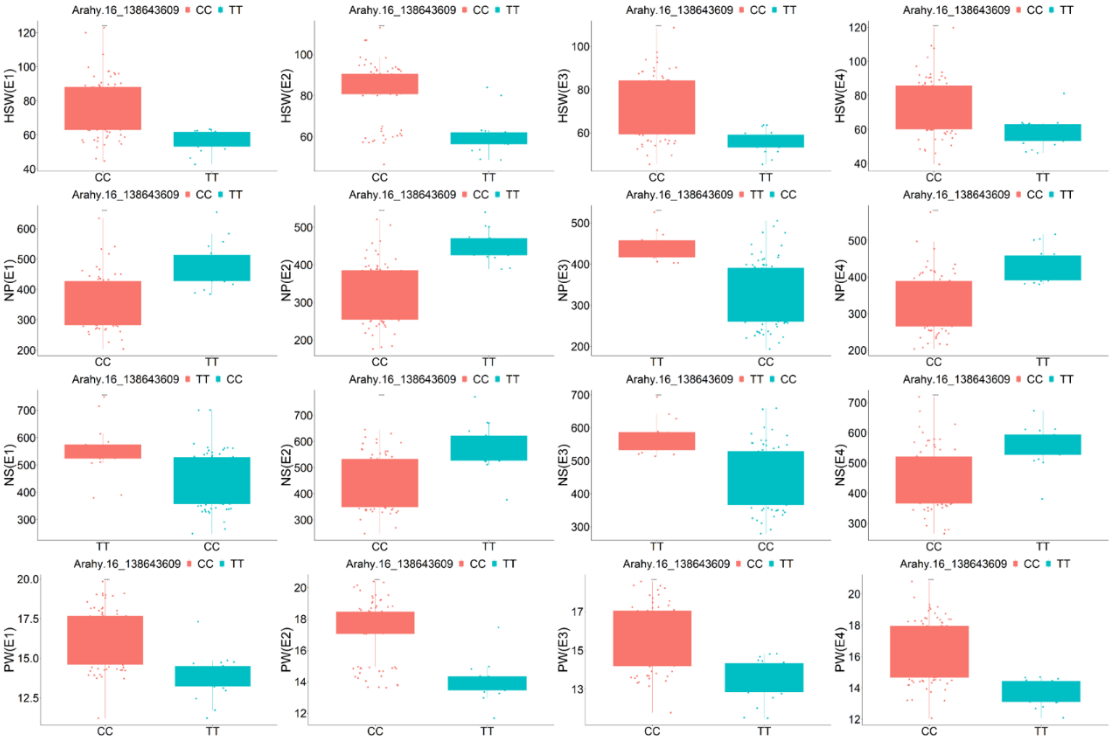
Arahy.16_138643609 CC TT
HSW(E1)
HSW(E2)
HSW(E3)
HSW(E4)
Arahy.16_138643609 CC TT
NP(E1)
NP(E2)
Arahy.16_138643609 TT CC
NP(E3)
NP(E4)
Arahy.16_138643609 TT CC
NS(E1)
Arahy.16_138643609 CC TT
NS(E2)
Arahy.16_138643609 TT CC
NS(E3)
Arahy.16_138643609 CC TT
NS(E4)
Arahy.16_138643609 CC TT
PW(E1)
PW(E2)
PW(E3)
PW(E4)

**B**

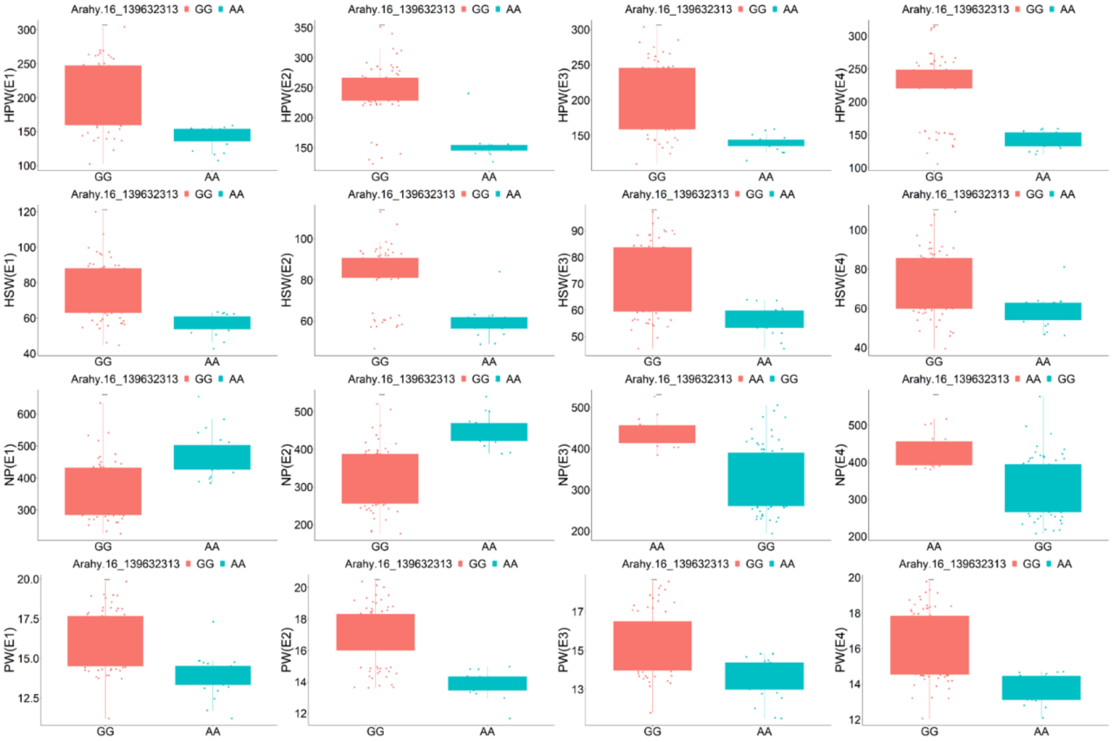
Arahy.16_139632313 GG AA
HPW(E1)
HPW(E2)
HPW(E3)
HPW(E4)
Arahy.16_139632313 GG AA
HSW(E1)
HSW(E2)
HSW(E3)
HSW(E4)
Arahy.16_139632313 GG AA
NP(E1)
NP(E2)
Arahy.16_139632313 AA GG
NP(E3)
NP(E4)
Arahy.16_139632313 GG AA
PW(E1)
PW(E2)
PW(E3)
PW(E4)

C

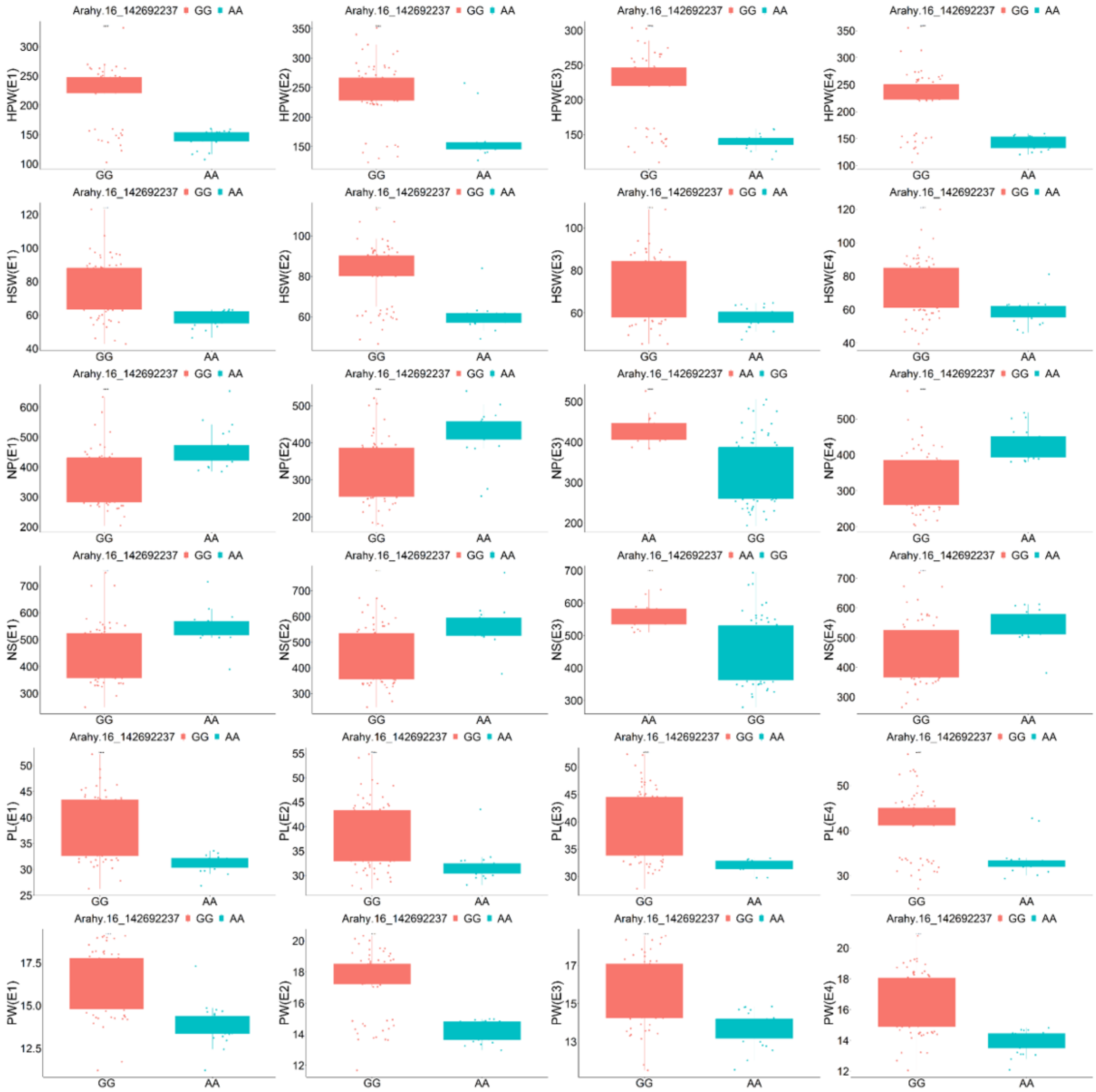
Arahy.16_142692237 GG AA
HPW(E1)
HPW(E2)
HPW(E3)
HPW(E4)
HSW(E1)
HSW(E2)
HSW(E3)
HSW(E4)
NP(E1)
NP(E2)
Arahy.16_142692237 AA GG
NP(E3)
NP(E4)
NS(E1)
NS(E2)
NS(E3)
NS(E4)
PL(E1)
PL(E2)
PL(E3)
PL(E4)
PW(E1)
PW(E2)
PW(E3)
PW(E4)
GG
AA

D

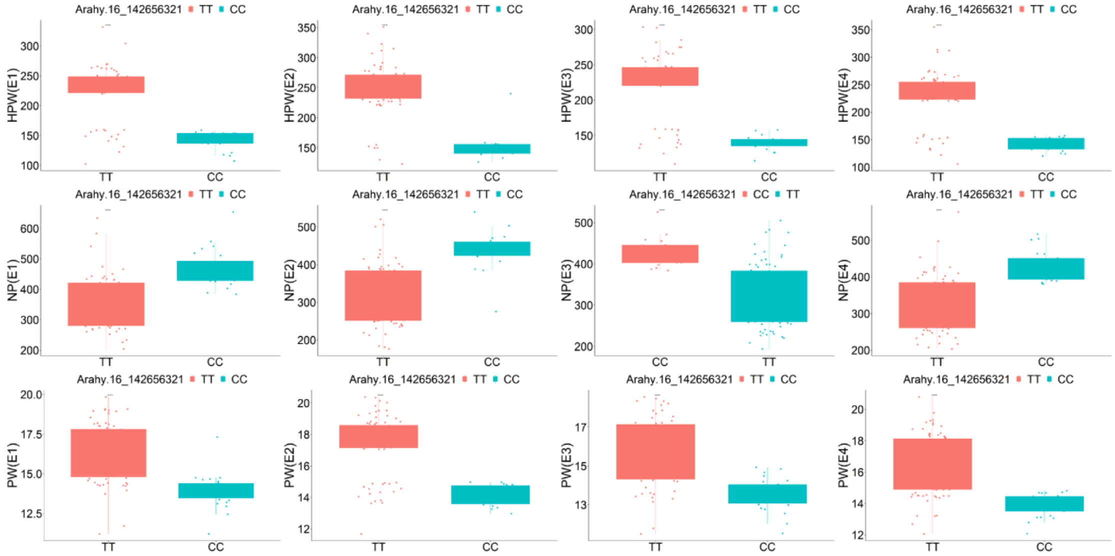
Arahy.16_142656321 TT CC
HPW(E1)
HPW(E2)
HPW(E3)
HPW(E4)
NP(E1)
NP(E2)
Arahy.16_142656321 CC TT
NP(E3)
NP(E4)
PW(E1)
PW(E2)
PW(E3)
PW(E4)
TT
CC

**E**

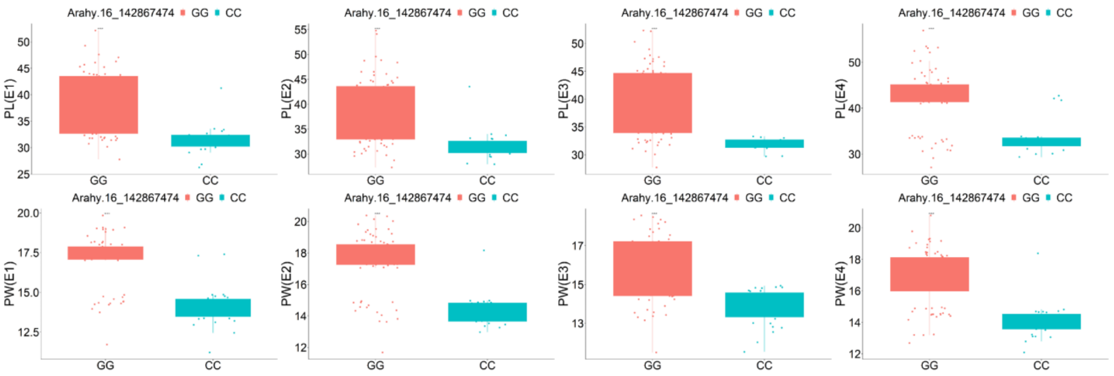


**F**

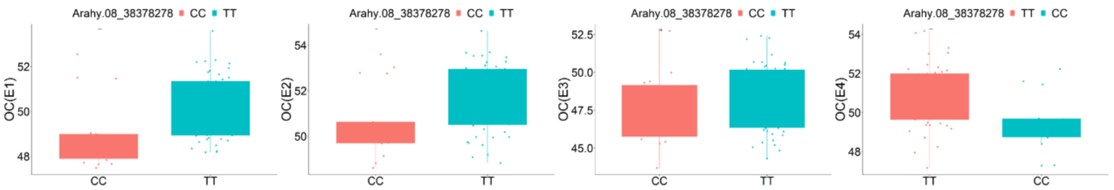


**G**

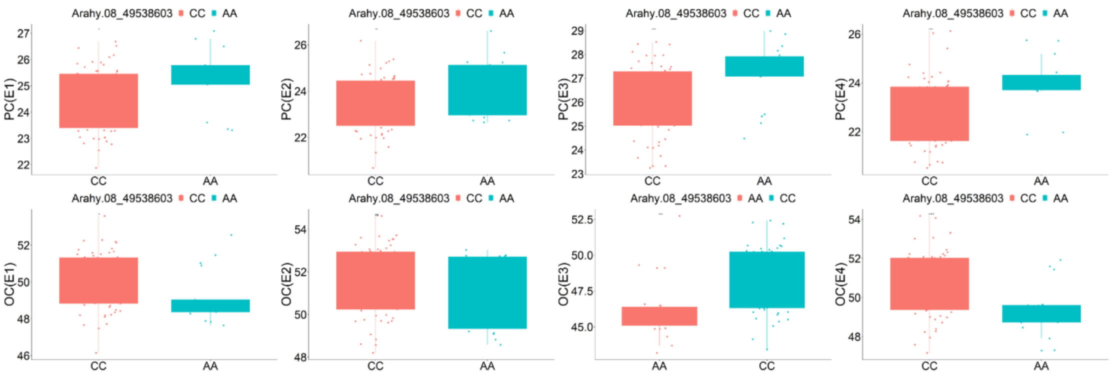


**Fig. S4.** Phenotypic difference between two base types at seven significant SNPs. A-G: Phenotypic difference between two base types at Arahy.16_138643609, Arahy.16_139632313, Arahy.16_142692237, Arahy.16_142656321, Arahy.16_142867474, Arahy.08_38378278, and Arahy.08_49538603. HPW, hundred-pod weight; HSW, hundred-seed weight; NP, total number of 500 grams of pods; NS, total number of 250 grams of seeds; PL, pod length; PW, pod width. PC, protein content; OC, oil content. E1, Kaifeng in 2019; E2, Xinyang in 2019; E3, Kaifeng in 2020; E4, Kaifeng in 2021.
