# Supplementary material for: Genome-wide association study and development of molecular markers for yield and quality traits in peanut (Arachis hypogaea L.)
Source: BMC Plant Biol. 2024 Apr 5;24:244. doi: 10.1186/s12870-024-04937-5 (PMC10996145; doi:10.1186/s12870-024-04937-5)
Supplement: Supplementary file 5 — Supplementary Material 5 [file 12870_2024_4937_MOESM5_ESM.pdf]

A

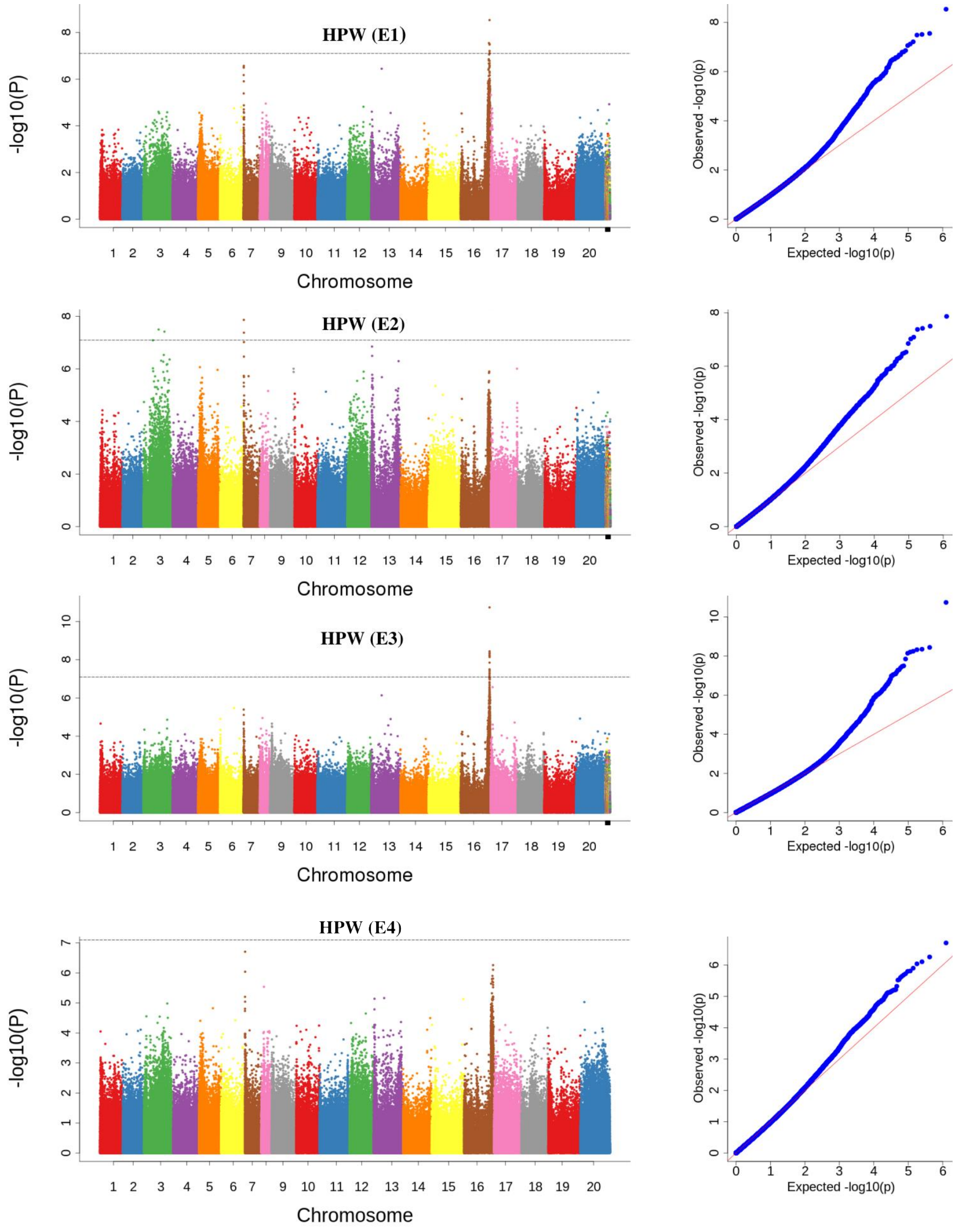
HPW (E1)
HPW (E2)
HPW (E3)
HPW (E4)
-log10(P)
Chromosome
1 2 3 4 5 6 7 9 10 11 12 13 14 15 16 17 18 19 20
Observed -log10(p)
Expected -log10(p)

B

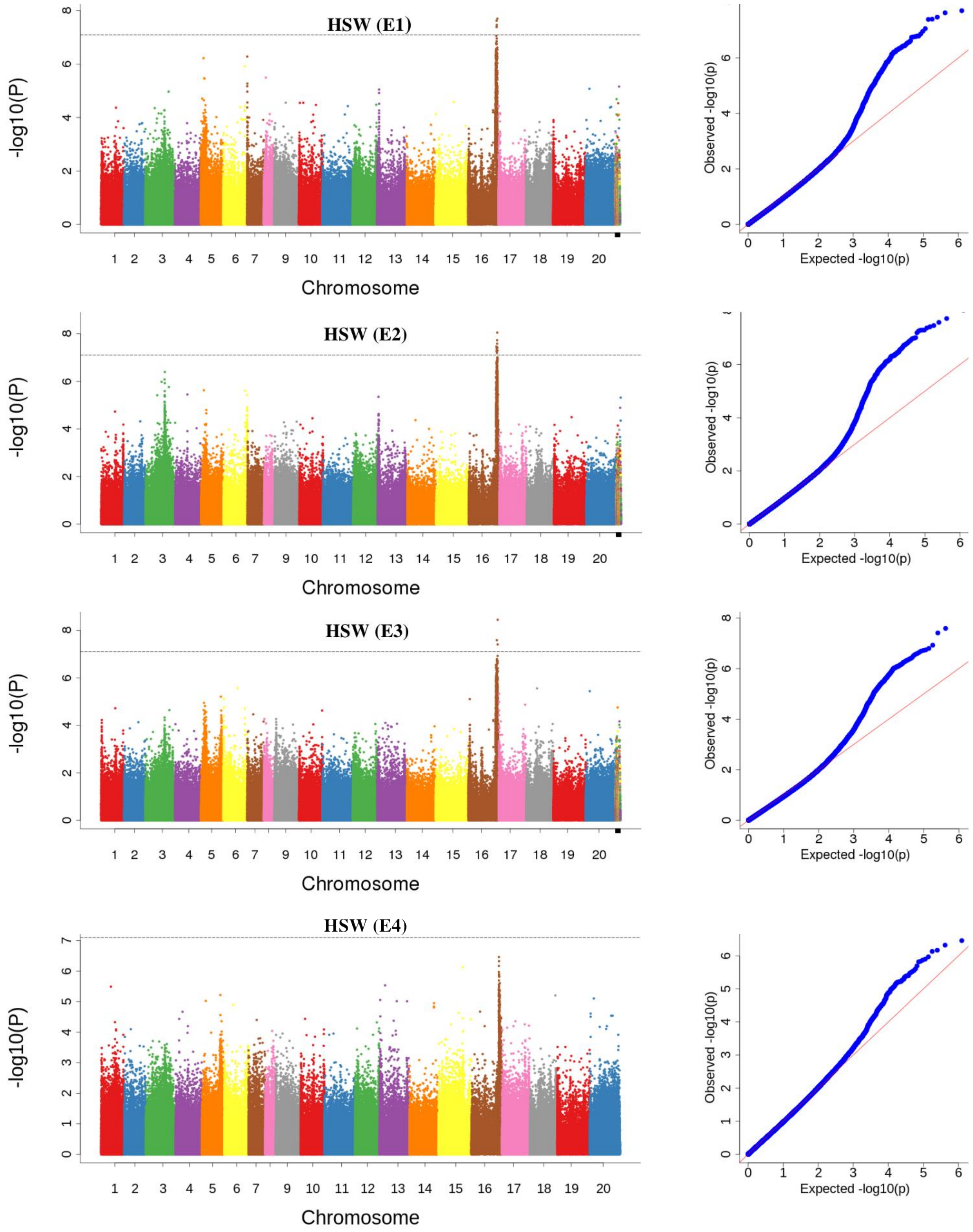
HSW (E1)
HSW (E2)
HSW (E3)
HSW (E4)
-log10(P)
Chromosome
1 2 3 4 5 6 7 9 10 11 12 13 14 15 16 17 18 19 20
Observed -log10(p)
Expected -log10(p)

C

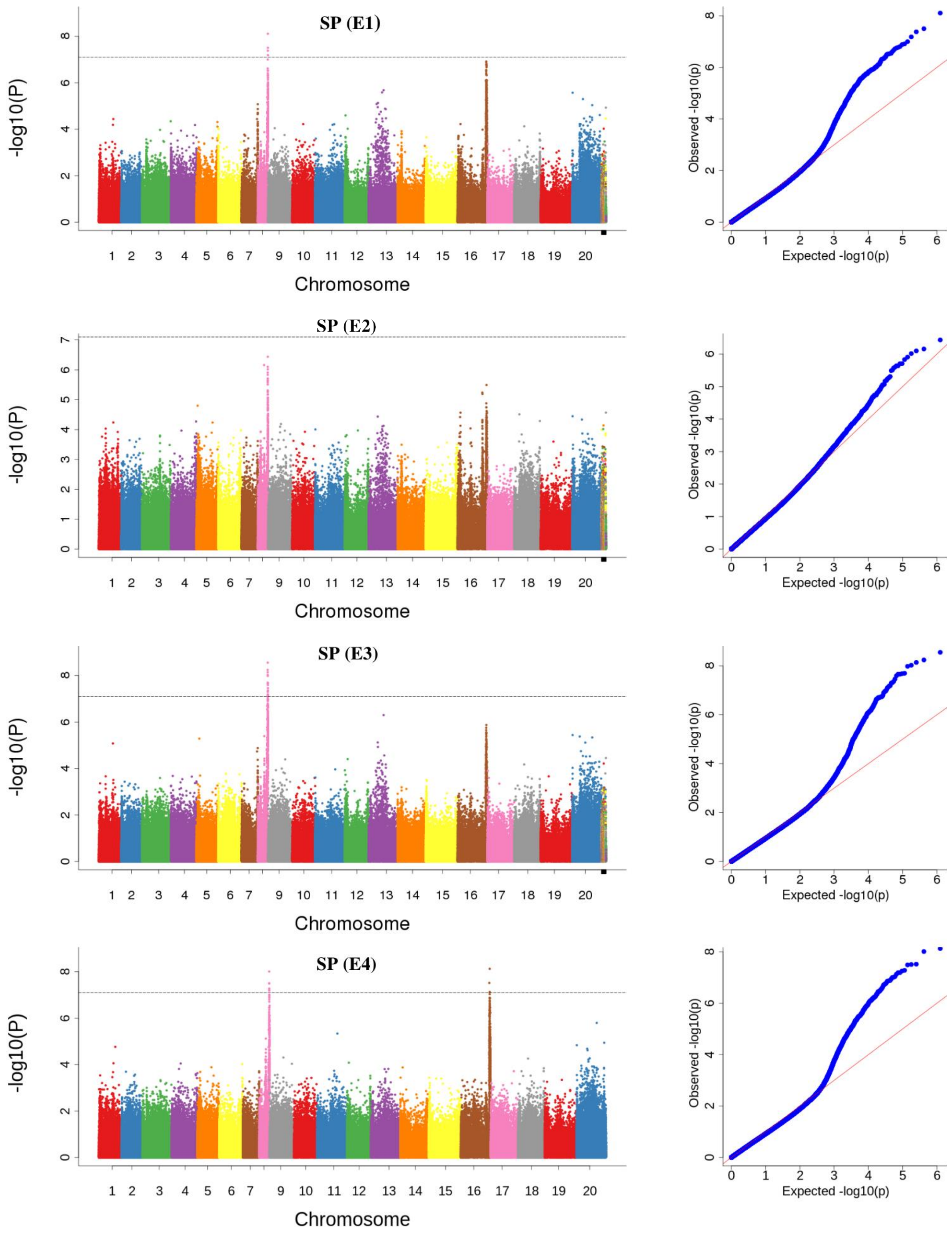
SP (E1)
SP (E2)
SP (E3)
SP (E4)
-log10(P)
Chromosome
Observed -log10(p)
Expected -log10(p)

D

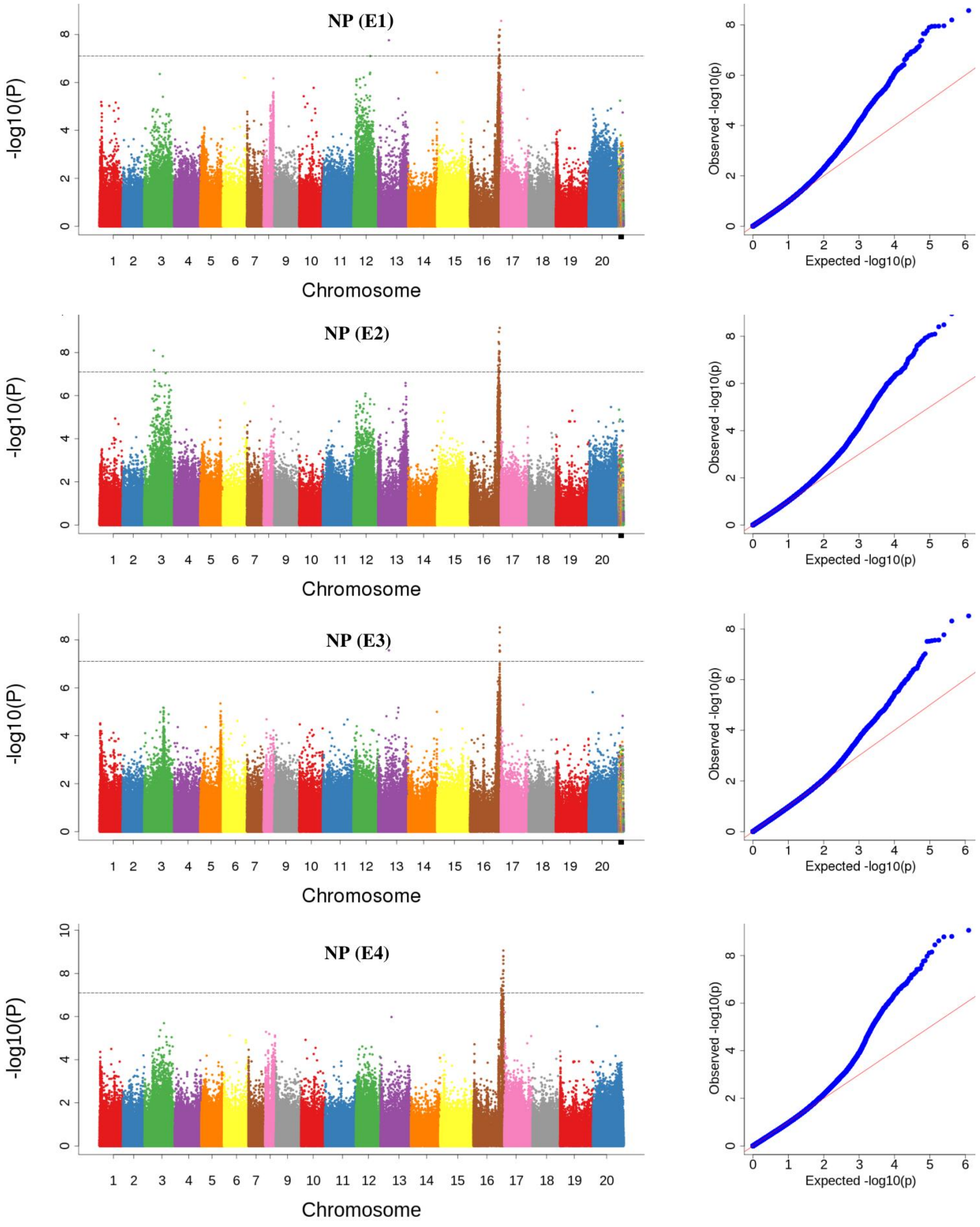

NP (E1)
NP (E2)
NP (E3)
NP (E4)
-log10(P)
Chromosome
1 2 3 4 5 6 7 9 10 11 12 13 14 15 16 17 18 19 20
Observed -log10(p)
Expected -log10(p)

E

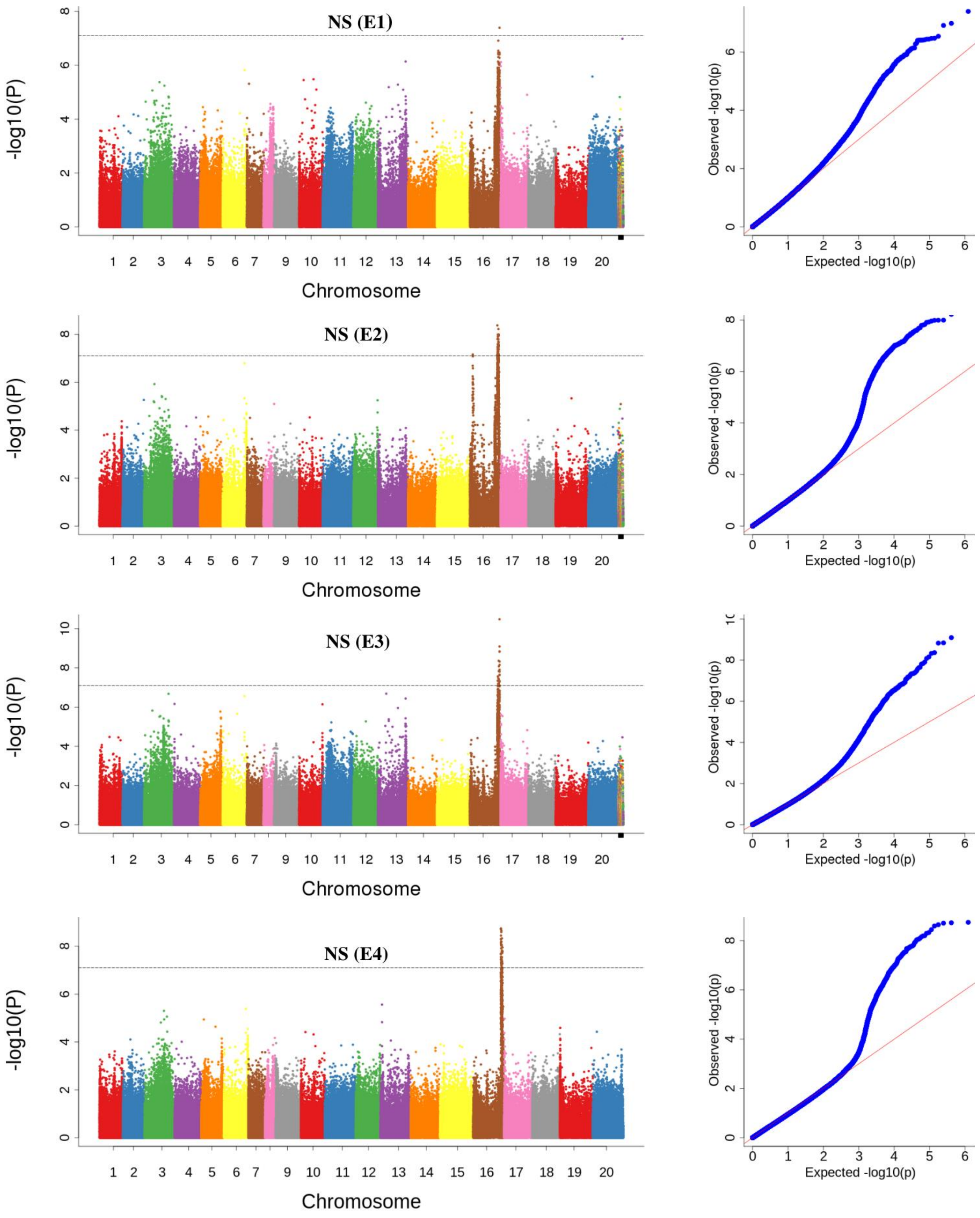
NS (E1)
NS (E2)
NS (E3)
NS (E4)
-log10(P)
Chromosome
Observed -log10(p)
Expected -log10(p)

F

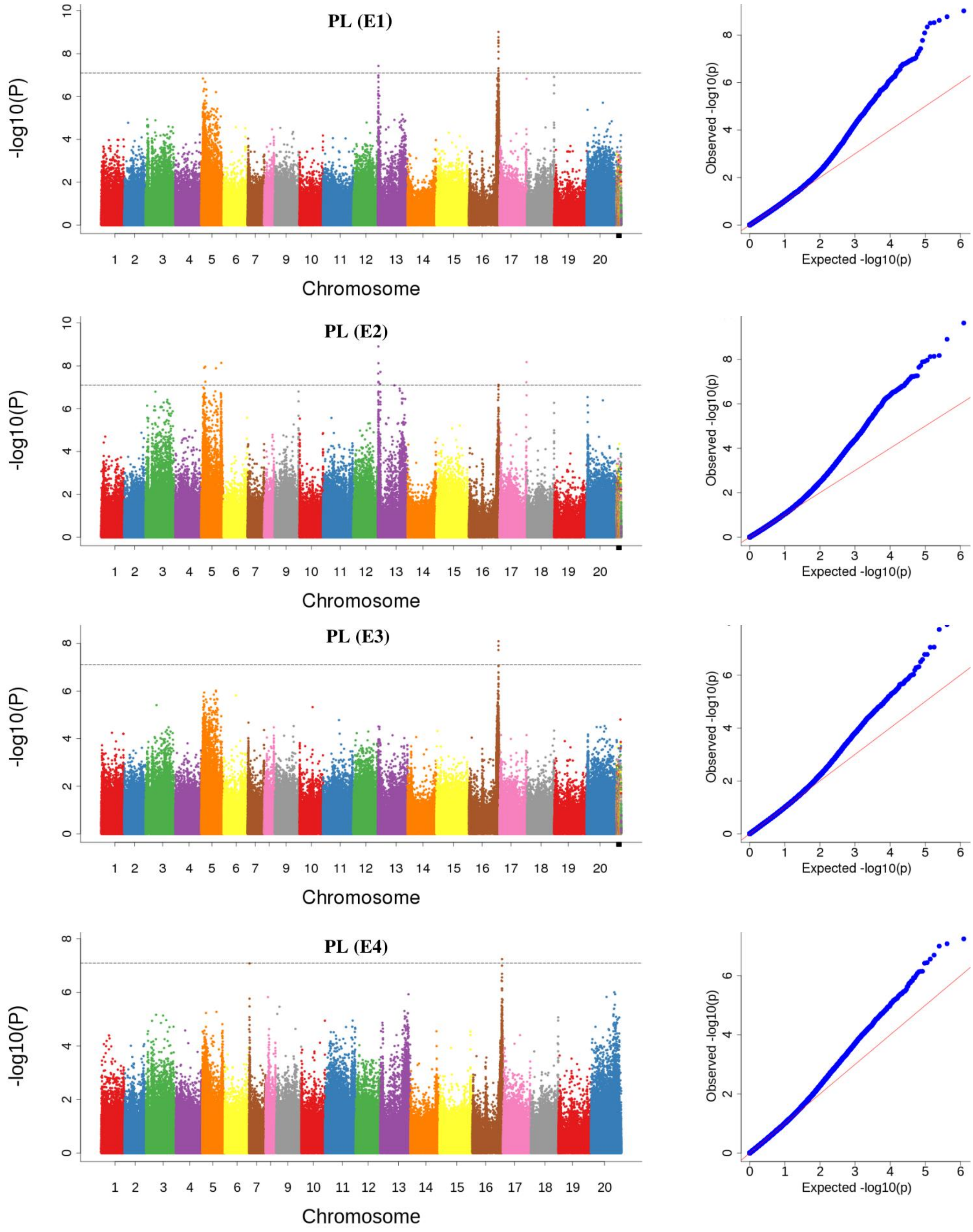
PL (E1)
PL (E2)
PL (E3)
PL (E4)
-log10(P)
Chromosome
1 2 3 4 5 6 7 9 10 11 12 13 14 15 16 17 18 19 20
Observed -log10(p)
Expected -log10(p)

G

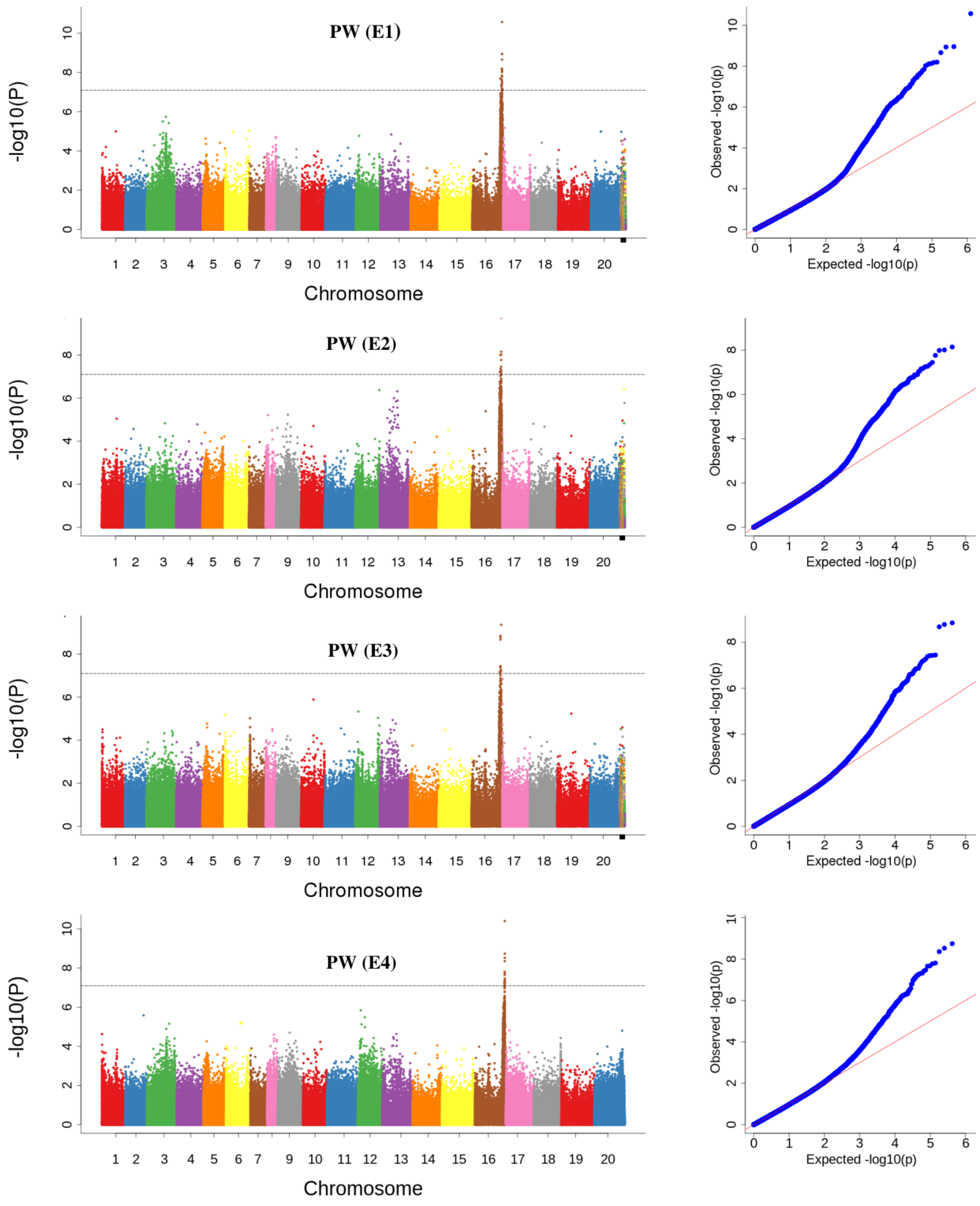
PW (E1)
PW (E2)
PW (E3)
PW (E4)
-log10(P)
Chromosome
1 2 3 4 5 6 7 9 10 11 12 13 14 15 16 17 18 19 20
Observed -log10(p)
Expected -log10(p)

H

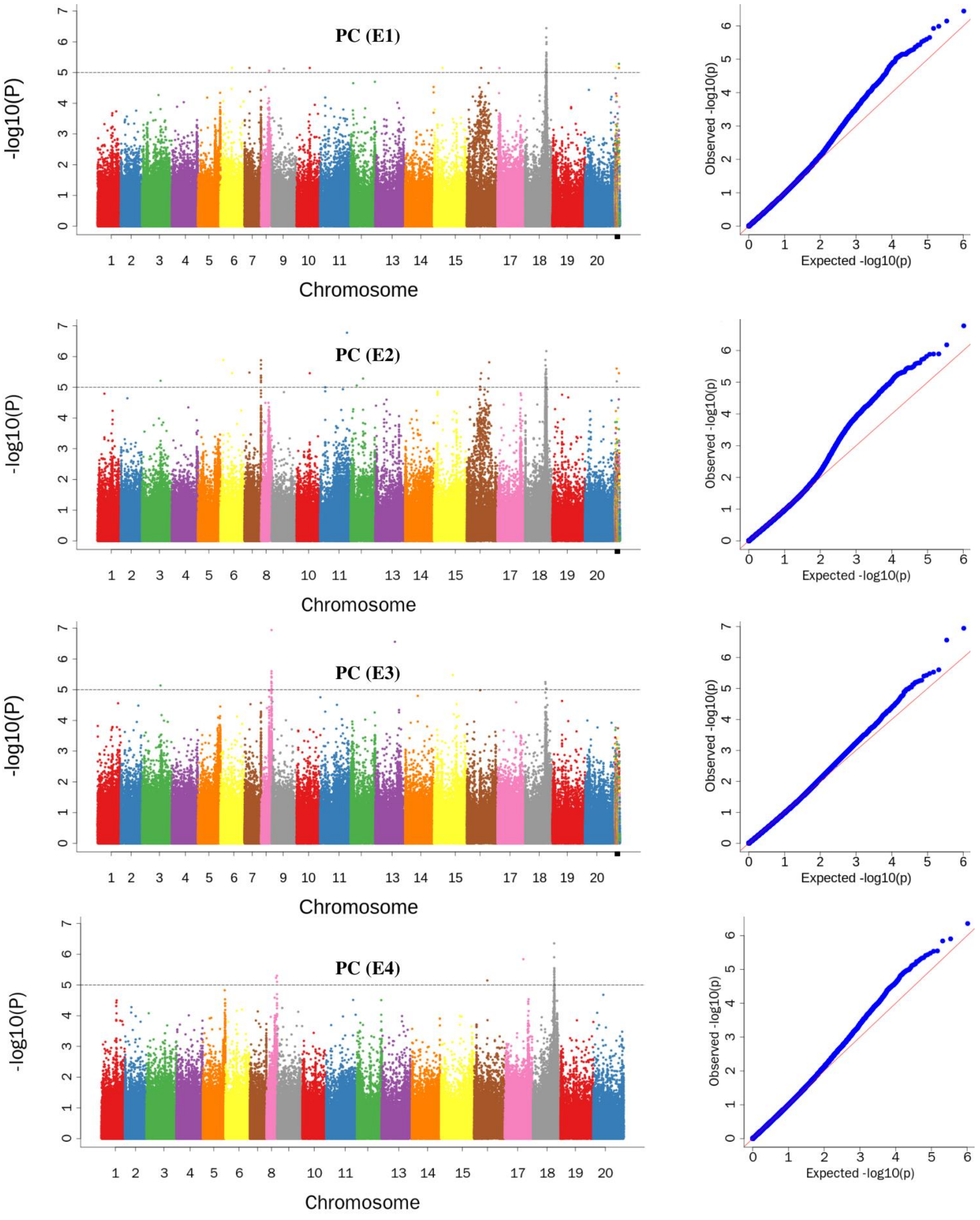
PC (E1)
PC (E2)
PC (E3)
PC (E4)
-log10(P)
Chromosome
Observed -log10(p)
Expected -log10(p)

I

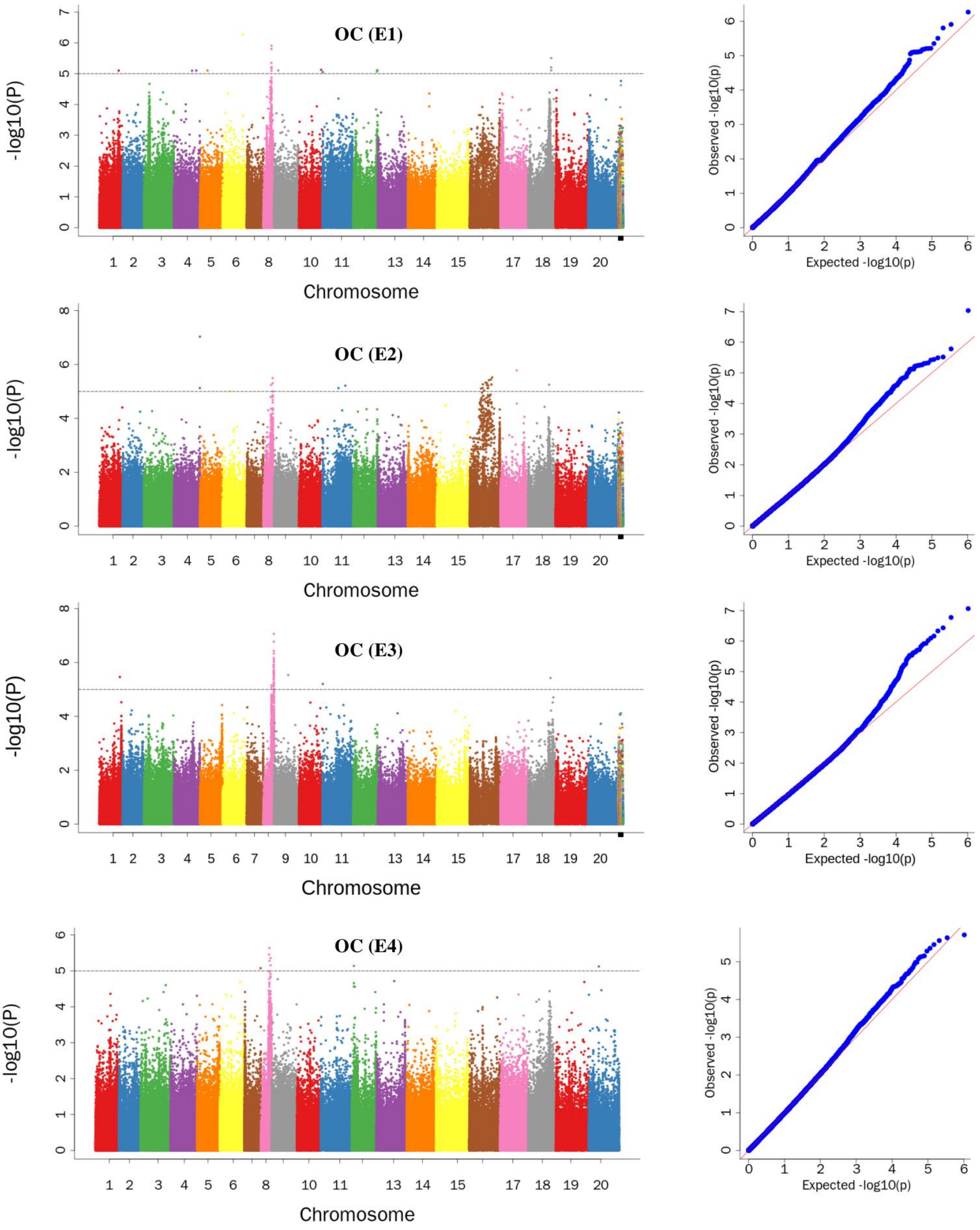

Fig. S3. Manhattan plot and quantile-quantile plot for yield and quality traits in four environments. A.

Manhattan plot and quantile-quantile plot for HPW in four environments; B. Manhattan plot and quantile-quantile plot for HSW in four environments; C. Manhattan plot and quantile-quantile plot for SP in four environments; D. Manhattan plot and quantile-quantile plot for NP in four environments; E. Manhattan plot and quantile-quantile plot for NS in four environments; F. Manhattan plot and quantile-quantile plot for PL in four environments; G. Manhattan plot and quantile-quantile plot for PW in four environments; H. Manhattan plot and quantile-quantile plot for PC in four environments; I. Manhattan plot and quantile-quantile plot for OC in four environments. The significance threshold of -log 10 (P) value is noted with the horizontal line. HPW, hundred-pod weight; HSW, hundred-seed weight; SP, shelling percentage; NP, total number of 500 grams of pods; NS, total number of 250 grams of seeds; PL, pod length; PW, pod width; PC, protein content; OC, oil content.; E1, Kaifeng in 2019; E2, Xinyang in 2019; E3, Kaifeng in 2020; E4, Kaifeng in 2021.
