# Supplementary material for: Genome-wide association study and development of molecular markers for yield and quality traits in peanut (Arachis hypogaea L.)
Source: BMC Plant Biol. 2024 Apr 5;24:244. doi: 10.1186/s12870-024-04937-5 (PMC10996145; doi:10.1186/s12870-024-04937-5)
Supplement: Supplementary file 6 — Supplementary Material 6 [file 12870_2024_4937_MOESM6_ESM.pdf]

Table S4 Genome-wide significantly associated loci and peak value in LD block region.

Chr	LD region	Peak snp	Env	Trait							
				HPW	HSW	NP	NS	PL	PW	PC	OC
16	138532301-138756092	138643609	E1	-	7.63	7.90	-	-	-		
			E2	-	-	8.95	7.47	-	7.99		
			E3	-	7.59	-	8.36	-	8.84		
16	139532424-139731914	139632313	E1	7.55	7.47	7.65	-	-	7.37		
			E2	-	7.30	-	-	-	7.35		
			E3	-	-	-	8.08	-	8.77		
			E4	-	-	7.23	-	-	-		
16	142542242-142737244	142656321	E1	7.12	-	-	-	-	-		
			E2	8.24	-	7.58	-	-	7.16		
			E3	-	-	7.55	7.29	-	-		
			E4	-	-	9.06	-	-	8.35		
16	142578547-142795108	142692237	E1	8.53	7.70	-	7.39	-	10.57		
			E2	10.74	-	9.14	-	-	9.73		
			E3	-	8.45	8.52	10.48	7.70	9.36		
			E4	-	-	-	-	-	10.4		
16	142775097-142972005	142867474	E1	-	-	7.53	-	9.02	8.06		
			E2	8.14	-	-	-	7.11	7.17		
			E3	-	-	-	-	8.09	-		
			E4	-	-	-	-	7.25	7.28		
8	3827767-38491489	38378278	E1							5.06	5.91
8	49428114-49646678	49538603	E3							6.94	7.06
			E4							5.30	-

HPW, hundred-pod weight; HSW, hundred-seed weight; NP, total number of 500 grams of pods; NS, total number of 250 grams of seeds; PL, pod length; PW, pod width; PC, protein content; OC, oil content.

E1, Kaifeng in 2019; E2, Xinyang in 2019; E3, Kaifeng in 2020; E4, Kaifeng in 2021.
