# Supplementary material for: Genome-wide association study and development of molecular markers for yield and quality traits in peanut (Arachis hypogaea L.)
Source: BMC Plant Biol. 2024 Apr 5;24:244. doi: 10.1186/s12870-024-04937-5 (PMC10996145; doi:10.1186/s12870-024-04937-5)
Supplement: Supplementary file 7 — Supplementary Material 7 [file 12870_2024_4937_MOESM7_ESM.pdf]

Table S2 The detailed information of SNP for nine traits.

Trait	E1<br>snp_num	E2<br>snp_num	E3<br>snp_num	E4<br>snp_num	No. of SNP	No. of repeatly detected SNP in different enviroments
HPW	4	4	7	0	12	3
HSW	5	11	2	0	14	4
SP	3	0	14	8	18	7
NP	12	17	5	14	37	9
NS	1	39	21	41	84	15
PL	6	17	3	1	23	2
PW	20	10	9	13	32	13
PC	27	39	14	16	82	12
OC	17	24	24	8	72	1
Total	95	161	99	101	374	66

HPW, hundred-pod weight; HSW, hundred-seed weight; SP, Shelling percentage; NP, total number of 500 grams of pods; NS, total number of 250 grams of seeds; PL, pod length; PW, pod width; PC, protein content; OC, oil content. E1, Kaifeng in 2019; E2, Xinyang in 2019; E3, Kaifeng in 2020; E4, Kaifeng in 2021.
