# Supplementary material for: Genome-wide association study and development of molecular markers for yield and quality traits in peanut (Arachis hypogaea L.)
Source: BMC Plant Biol. 2024 Apr 5;24:244. doi: 10.1186/s12870-024-04937-5 (PMC10996145; doi:10.1186/s12870-024-04937-5)
Supplement: Supplementary file 8 — Supplementary Material 8 [file 12870_2024_4937_MOESM8_ESM.pdf]

Table S5 The primer sequence of KASP markers.

SNP	Primer (5' to 3')
Arahy.16_142682809	GAAGGTGACCAAGTTCATGCTACAACAATTCACATATCATCCAACTCAAA
	GAAGGTCGGAGTCAACGGATTAACAATTCACATATCATCCAACTCAAC
	GGTGGGGTTATAATTATTATTATTATAAGCTAAG
Arahy.08_38352339	GAAGGTGACCAAGTTCATGCTCGTCCCGGTTGCCTTCCAG
	GAAGGTCGGAGTCAACGGATTCCGTCCCGGTTGCCTTCCAT
	CGGCCAAACAGGGCGGCAAC
Arahy.08_49440731	GAAGGTGACCAAGTTCATGCTCATACTTGCGCAAGAAATTAGCGG
	GAAGGTCGGAGTCAACGGATTCCATACTTGCGCAAGAAATTAGCGA
	AAACACCAGTATGACTGGGATGCTG
