# Supplementary material for: Genome-wide association study and development of molecular markers for yield and quality traits in peanut (Arachis hypogaea L.)
Source: BMC Plant Biol. 2024 Apr 5;24:244. doi: 10.1186/s12870-024-04937-5 (PMC10996145; doi:10.1186/s12870-024-04937-5)
Supplement: Supplementary file 9 — Supplementary Material 9 [file 12870_2024_4937_MOESM9_ESM.pdf]

Table S1 ANOVA for nine traits in four environments.

Source Variation	df	HPW	HSW	SP	NP	NS	PL	PW	PC	OC
Env	3	175.80**	88.54**	572.85**	151.99**	4.79**	133.79**	116.96**	2115.07**	1718.81**
G	198	64.97**	47.77**	47.99**	50.40**	35.93**	78.52**	28.03**	17.28**	18.47**
G × Env	594	2.05**	1.51**	1.99**	1.70**	1.81**	1.68**	1.13*	1.91**	2.42**
Env × Rep	6	74.21**	26.98**	111.47**	81.01**	97.23**	34.00**	2.47*	26.98**	49.29**

Env, environment; G, Genotype; Rep, replication. **, significant at $p < 1\%$ level, *, significant at $p < 5\%$ level.
