# Supplementary material for: Genome-wide association study and development of molecular markers for yield and quality traits in peanut (Arachis hypogaea L.)
Source: BMC Plant Biol. 2024 Apr 5;24:244. doi: 10.1186/s12870-024-04937-5 (PMC10996145; doi:10.1186/s12870-024-04937-5)
Supplement: Supplementary file 10 — Supplementary Material 10 [file 12870_2024_4937_MOESM10_ESM.pdf]

Table S3 The SNPs detected to be associated with multiple traits.

Chr.	Position	Ref	Alt	Trait (No. of environment detected the signal)
8	38378278	C	T	PC(1), OC(1)
8	44879304	C	T	PC(1), OC(1)
8	48994448	T	C	PC(1), OC(1)
8	49074383	A	G	SP(2), OC(1)
8	49104846	A	G	SP(2), OC(1)
8	49296302	A	G	PC(1), OC(1)
8	49338631	A	G	PC(1), OC(1)
8	49385915	T	C	PC(1), OC(1)
8	49428114	T	A	SP(2), OC(1)
8	49538603	A	C	PC(2), OC(1)
8	49587942	T	C	PC(1), OC(1)
8	50246048	T	C	SP(2), OC(1)
16	104322766	G	T	PC(1), OC(1)
16	107768318	G	T	PC(1), OC(1)
16	132149114	T	C	NP(1), NS(1)
16	132715371	C	T	NP(1), NS(2)
16	134192728	C	T	HSW(1), NS(2)
16	134442558	T	C	NP(1), NS(2)
16	134838333	G	A	HSW(1), NS(2)
16	134885227	C	T	HSW(1), NS(2)
16	134917536	A	G	NP(1), NS(1)
16	136394778	G	A	HSW(1), NS(1)
16	137019564	A	C	HSW(1), NS(1)
16	137041249	A	G	NP(2), NS(3)
16	137224495	G	A	HSW(1), NS(1)
16	137375522	A	G	NP(1), NS(1)
16	137415227	C	T	HSW(1), NS(3)
16	137588421	G	C	NP(1), PW(1)
16	137742110	C	T	HSW(1), NS(1)
16	138494143	A	G	NP(1), NS(1)
16	138643609	C	T	HSW(2), NP(2), NS(2), PW(2)
16	138768362	T	C	HSW(2), NS(2)
16	139088298	A	G	HSW(1), NP(1)
16	139140826	G	A	NP(2), NS(3), PW(1)
16	139438661	C	T	NP(2), NS(2), PW(1)
16	139632313	G	A	HPW(1), HSW(2), NP(2), NS(1), PW(3)
16	139638736	G	A	NP(1), NS(1)
16	140670972	G	A	NP(1), PW(2)
16	142529705	C	T	HPW(1), NS(1)
16	142588343	C	T	SP(1), PW(1)
16	142641021	A	G	PL(1), PW(1)
16	142656321	C	T	HPW(2), NP(3), NS(1), PW(2)

16	142692237	G	A	HPW(2), HSW(2), NP(2), NS(2), PL(1), PW(4)
16	142737054	G	A	HPW(1), SP(1), PW(3)
16	142775091	C	T	HPW(1), NP(3), NS(1), PW(3)
16	142815688	G	A	HPW(2), NP(1), PL(1), PW(3)
16	142867474	C	G	HPW(1), NP(1), PL(4), PW(3)
16	142913778	G	A	NP(1), NS(1)

HPW, hundred-pod weight; HSW, hundred-seed weight; SP, Shelling percentage; NP, total number of 500 grams of pods; NS, total number of 250 grams of seeds; PL, pod length; PW, pod width; PC, protein content; OC, oil content.
